# Unravelling the role of Sildenafil and SB204741 in suppressing fibrotic potential of peritoneal fibroblasts obtained from PD patients

**DOI:** 10.3389/fphar.2023.1279330

**Published:** 2024-01-23

**Authors:** Saurabh Chaturvedi, Harshit Singh, Vikas Agarwal, Akhilesh Jaiswal, Narayan Prasad

**Affiliations:** ^1^ Department of Clinical Immunology and Rheumatology, Sanjay Gandhi Post Graduate Institute of Medical Sciences, Lucknow, Uttar Pradesh, India; ^2^ Department of Medical Laboratory Technology, School of Allied Health Sciences, Delhi Pharmaceutical Sciences and Research University, New Delhi, India; ^3^ Immuno Biology Lab, Translational Health Science and Technology Institute, Faridabad, Haryana, India; ^4^ Department of Nephrology and Renal Transplantation, Sanjay Gandhi Post Graduate Institute of Medical Sciences, Lucknow, Uttar Pradesh, India

**Keywords:** peritoneal fibrosis, serotonin, TGF-β1, sildenafil, SB204741, ACTA2, peritoneal dialysis inflammation

## Abstract

**Introduction:** Peritoneal fibrosis (PF) results in technique failure in peritoneal dialysis (PD) patients. Peritoneal fibroblasts are characterized by increase in the ACTA2 gene, responsible for alpha smooth muscle actin (α−SΜΑ), extracellular matrix (ECM) production, and inflammatory cytokines production, which are the are key mediators in the pathogenesis of PF. 5-hydroxytryptamine (5-HT; serotonin) induces ECM synthesis in fibroblasts in a transforming growth factor-beta 1 (TGF-β1) dependent manner. The purpose of our study was to identify the potential mechanism and role of sildenafil and 5HT_2B_ receptor inhibitor (SB204741) combination in attenuating PD-associated peritoneal fibrosis.

**Methods:** Studies were performed to determine the effect of TGF-β1, sildenafil, and SB204741 on human peritoneal fibroblasts (HPFBs) isolated from the parietal peritoneum of patients in long-term PD patients (n = 6) and controls (n = 6). HPFBs were incubated with TGF-β1 (10 ng/mL) for 1 h and later with TGF-β1 (10 ng/mL)/[sildenafil (10 µM) or SB204741 (1 µM)] and their combination for 24 h (post-treatment strategy). In the pre-treatment strategy, HPFBs were pre-treated with sildenafil (10 µM) or SB204741 (1 µM) and a combination of the two for 1 h and later with only TGF-β1 (10 ng/mL) for 24 h.

**Results:** The anti-fibrotic effects of the combination of sildenafil and SB204741 were greater than that of each drug alone. In TGF-β1-stimulated HPFBs, pro-fibrotic genes (*COL1A1, COL1A2, ACTA2, CTGF*, *FN1,* and *TGFB1*) exhibited higher expression than in controls, which are crucial targets of sildenafil and SB204741 against peritoneal fibrosis. The synergistic approach played an anti-fibrotic role by regulating the pro- and anti-fibrotic gene responses as well as inflammatory cytokine responses. The combination treatment significantly attenuated peritoneal fibrosis, as evident by the almost complete amelioration of *ACTA2* expression, restoration of anti-fibrotic genes (*MMP2/TIMP1*), and, at least, by reducing the expression of pro-inflammatory cytokines (IFN-γ, IL-4, IL-17, IL-1β, IL-6, TNF-α, and TGF-β1) along with an increase in IL-10 levels.

**Discussion:** Taken together, the above research evidences that the combination of sildenafil and SB204741 may have therapeutic potential in suppressing peritoneal fibrosis due to peritoneal dialysis.

## Introduction

Peritoneal dialysis (PD) is an established modality of treatment for end-stage renal disease (ESRD) patients (Grassmann et al., 2005). The development of high transporter membranes, ultrafiltration failure (UFF), and, subsequently, peritoneal fibrosis (PF) is one of the most common causes of technique failure in PD patients in the long term. The persistent inflammatory changes due to exposure to a bio-incompatible solution lead to peritoneal myofibroblast (PMFB) formation and fibrosis, leading to reduced solute clearance and UFF, an adverse effect of the treatment on long-term PD patients. Despite several advances and mechanisms explored in the pathogenesis of PF, the therapeutic approaches to prevent or halt the development or progression of PF are not fully uncovered.

The key fibrogenic cytokine factor, transforming growth factor (TGF-β1), is responsible for progressive changes in the peritoneal mesothelial cells (PMCs) during PF (Gangji et al., 2009; Heo et al., 2021). One study on rodent peritoneum has reported that TGF-β1 induces epithelial-to-mesenchymal transition (EMT) similar to that observed in the peritoneal tissues of PD patients (Margetts et al., 2005). EMT involves changes in cell membrane receptors; signaling molecules such as TGF-β1, Src, and hypoxia-inducible factor (HIF); and cell morphology and behavior (Wilson et al., 2020). TGF-β1 and HIF signaling pathways activate fibroblasts in encapsulating peritoneal sclerosis (EPS) (Wilson et al., 2020). The importance of TGF-β1 signaling for EMT in PMCs has been demonstrated by using the TGF-β receptor inhibitor GW788388 in the EMT signaling pathway (Lho et al., 2021). The peritoneal membrane (PM) submesothelial compact zone undergoes progressive thickening due to PF, which is characterized by loss of PMCs, increased angiogenesis, enhanced myofibroblast proliferation, and abnormal extracellular matrix (ECM) protein deposition. These changes impair the function of the PM as a dialysis membrane. Mesothelial–mesenchymal transition (MMT) is a key mechanism that contributes to development of PF by generating fibroblasts and myofibroblasts from PMCs (Huang et al., 2023). MMT is a physiological process that generates fibroblasts and related cells from mesothelial cells to repair damaged tissues. This process is normally self-limiting and stops when the injury is resolved. However, under persistent stimuli, MMT can become pathological and lead to excessive fibroblast proliferation and tissue fibrosis (Huang et al., 2023). TGF-β1 is involved in the differentiation of PMFBs of resident fibroblasts and also in the mesenchymal conversion of endothelial cells via endothelial-to-mesenchymal transition (EnMT), which call forth to target vasculopathy for the abrogation of fibrosis (Zeisberg et al., 2007; Zeisberg et al., 2008; Li et al., 2009; Beyer et al., 2012).

Nitric oxide (NO), a potent vasodilator, mediates its action by NO/soluble guanylate cyclase (sGC)/cyclic GMP (cGMP)/protein kinase G (PKG) signaling (Palmer et al., 1988; Takimoto, 2012). Cyclic nucleotide phosphodiesterases (PDEs) are crucial components in the cyclic adenosine monophosphate/protein kinase A (cAMP/PKA) and cGMP-PKG signaling pathways. PDEs hydrolyze phosphodiester bonds of cAMP and cGMP into the linear and inactive form (Kniotek and Boguska, 2017). PDE5 selective competitive inhibitors, sildenafil and tadalafil, have shown excellent outcomes in the treatment of systemic sclerosis (SSc)-related digital ulcers and pulmonary arterial hypertension (PAH) (Galiè et al., 2005; Ramani and Park, 2010). Hence, various PDE5 selective inhibitors have been reported to have anti-fibrotic effects in models of other fibrotic disorders (Ferrini et al., 2006).

Earlier studies also showed that 5-HT signaling shares a strong relationship with the tumor, inflammation, and fibrosis of the liver, lungs, and skin (Mann and Oakley, 2013; Wan et al., 2020). Elevated levels of whole blood 5-HT in chronic kidney disease (CKD) patients on dialysis therapy, especially PD, have been reported (Kerr et al., 1992). Intra-abdominal adhesion formation is regulated by peripheral serotonin, which acts through the 5-HT_2B_ receptor in the adhesive tissues. This receptor mediates the effects of serotonin on inflammation, oxidative stress, fibrinolytic system dysfunction, angiopoiesis, and TGF-β1 expression, which are all involved in the pathogenesis of adhesions (Bi et al., 2017). Cellular effects of 5-HT are mediated by seven different 5-HT receptor subtypes (5-HTR_1_–HTR_7_) (Hoyer et al., 2002) and among the class 2 (5-HT_2_) receptors, mainly subtype 5-HT_2A_ and 5-HT_2B_. In renal mesangial cells, 5-HT is known to potently activate extracellular signal-regulated kinases (ERK1/2) as well as TGF-β1 induction (Grewal et al., 1999). Numerous studies have demonstrated TGF-β1 as the main mediator of PF as well as its association with peritoneal membrane injury in PD patients (Loureiro et al., 2011; Yoshizawa et al., 2015; Wang et al., 2017). In a previous study, SSc treatment with 5-HT_2_/5-HT_2B_ receptor antagonists has been reported to reverse the attenuation of 5-HT-associated gene expression and collagen production (Dees et al., 2011). The role of 5-HT/TGF-β1/Smad3 signaling in the development of renal, liver, and cardiac fibrosis has been well-documented (Kim et al., 2018).

Several studies have stated a strong association between 5-HT and T effector cells, i.e., T helper (Th) 1, Th2, and Th17, as well as T regulatory cells (Tregs) (Wan et al., 2020). In experimental animal models of various inflammatory diseases, 5-HT is involved in the release of pro- and anti-inflammatory cytokines as well as in the imbalance of Th1, Th2, Th17, and Tregs (Wan et al., 2020). Th1 and Th2 cells differentially regulate fibrosis, and their respective cytokines play distinct roles in tissue remodeling and fibrosis (Wynn, 2008). In addition, Tregs play a critical role in maintaining immunological self-tolerance, and their immunoregulatory mechanisms have been robustly studied in severe inflammatory diseases (Miyara et al., 2014).

Taken together, the above studies and their findings instigated us to study the role of selective inhibitors of isoform 5 of the PDE enzyme and 5-HT_2B_ receptor individually as well as in combination for the abrogation of the fibrotic potential of HPFBs.

## Methods

### Patients and controls

In this prospectively designed study, we included peritoneal biopsy tissue obtained from ESRD patients on PD (n = 6) undergoing catheter replacement or removal after renal transplantation. All patients continued PD for at least 6 months before their inclusion in the study. All patients had been using a glucose-based solution (2.5% glucose Dianeal [Baxter, Deerfield, IL]) for three exchanges a day. The exclusion criteria for PD patients were historical peritonitis at the time of enrolment or 3 months prior to enrolment.

The peritoneal tissue for the control population of the study was taken from persons with normal renal function during laparoscopic donor nephrectomy after informed consent (n = 6). The control subjects did not have any history of severe pain abdomen, peritonitis, or surgery in the past.

A biopsy specimen of 4 mm^2^ in size was taken in normal saline (NS) and further processed in the laboratory for isolation of HPFBs. The present study complies with the Declaration of Helsinki. The study was approved by the Institutional Ethics Committee (IEC, human research) (document submission number: No: 2017-1-IMP-95), and informed written consent was obtained from all PD patients and controls. Demographic and clinical information of PD patients and controls is described in Table 1.

**TABLE 1:** Characteristics of the Patients and Controls.

Characteristics	Patients	Controls	*p*-value
Age (years)	46.50 ± 15.05	43.37 ± 15.52	0.712
Sex (Male)	5 (83.33%)	3 (50%)	-
Hemoglobin (gm/dL)	9.65 ± 0.77	13.85 ± 1.85	<0.001
S. creatinine (mg/dL)	7.52 ± 2.08	0.91 ± 0.27	0.001
S. BUN (mg/dL)	42.66 ± 8.02	8.25 ± 1.49	<0.001
S. calcium (mg/dL)	7.80 ± 0.89	9.23 ± 0.81	0.011
S. phosphorus (mg/dL)	4.48 ± 0.87	4.69 ± 0.19	0.595
S. albumin (g/dL)	2.65 ± 0.18	4.28 ± 0.68	<0.001
S. sodium (mmol/L)	139.67 ± 4.18	135.88 ± 6.01	0.189
S. potassium (mmol/L)	4.60 ± 0.51	3.92 ± 0.28	0.021
iPTH (pg/mL)	271.25 ± 53.45	-	-
CRP (mg/L)	4.67 ± 2.58	1.10 ± 0.62	0.019
Duration of PD (months)	23.67 ± 17.15	-	-
Infection during PD (times)	2.33 ± 1.21	-	-
Residual renal function (mL/min)	1.86 ± 1.41	6.52 ± 2.23	<0.001

iPTH, intact parathyroid hormone; CRP, C-reactive protein.

### Reagents

Dulbecco’s modified Eagle medium (DMEM, Cat No: D1152), sodium bicarbonate, sodium pyruvate, and trypsin-EDTA (Cat No: T4174) were purchased from Sigma, St Louis, MO, United States. The 100X Antibiotic–Antimycotic (Cat No: 15240062) and fetal bovine serum (FBS, Cat No: 10270106) were purchased from Gibco, Grand Island, NY, United States. Dimethyl sulfoxide (DMSO, Cat No: D2650) and [3-(4,5-dimethylthiazol-2-yl)-2, 5-diphenyltetrazolium bromide] (MTT, Cat No: M5655) was purchased from Sigma, St Louis, MO, United States. PDE-5 inhibitor sildenafil (Cat No: SML3033) was purchased from Sigma, United States. Serotonin, 5-HT (Cat No: 14927), and 5-HT_2B_ antagonist, SB204741 (Cat No: S0693) were purchased from Sigma, United States. Recombinant human TGF-β1 (Cat No: AF-100-21C) was purchased from PeproTech, United States. RNAiso Plus was purchased from Takara Bio Inc., Nojihigashi, Kusatsu, Japan. LightCycler^®^ 480 2X Maxima SYBR Green RT-PCR Master Mix was purchased from Roche Diagnostics, Indianapolis, IN, United States. The cDNA synthesis kit (Cat No: K1632) was purchased from Thermo Fisher Scientific Inc., Bartlesville, OK, United States. Anti-fibroblast FITC (Cat No: 130-100-135) was purchased from Miltenyi Biotec, Germany.

### Isolation and primary culture of HPFBs

The peritoneal biopsy tissue was washed with phosphate-buffered saline (PBS) two–three times. The tissue was then cut into small pieces and incubated overnight in a dispase solution (2.4 U/mL) at 37°C and 5% CO_2_ with shaking. After incubation, the remaining tissue was discarded, and the dispase solution was centrifuged to obtain cell pellets. The resulting pellet was transferred to a culture flask and incubated in Dulbecco’s modified Eagle medium (DMEM) with 10% fetal bovine serum (FBS) and 1% penicillin/streptomycin/amphotericin-B at 37°C and 5% CO_2_. The outgrowth of HPFBs occurred within 2 weeks (Witowski and Jörres, 2006), as shown in Supplementary Figure S1A. Isolation and culture of human peritoneal fibroblasts are depicted in Supplementary Figure S5. Cells were washed and plated in DMEM, and the cells between passages 3 to 5 were used for experiments (Chaturvedi et al., 2018).

### Flow cytometry analysis

Flow cytometry analysis was carried out to characterize the cell surface antigen of the fibroblasts. A minimum of 10,000 events were counted on FACSCanto™ II (Becton Dickinson, San Jose, CA). The stained cells were analyzed using FlowJo software v9.9.6 (Ashland, OR, United States; Supplementary Figure S1B).

### TGF-β1 induces expression of pro-fibrotic genes

HPFBs were stimulated with TGF-β1 at various concentrations to evaluate the effect on pro-fibrotic gene mRNA expression (Chaturvedi et al., 2018). The detailed methodology is described in Supplementary Method S1.

### MTT assay

The effect of the selective inhibitor of type 5 of the PDE enzyme (sildenafil) and 5-HT_2B_ receptor (SB204741) on the proliferative capacity of HPFBs was quantified using mitochondria-dependent reduction of a tetrazolium dye, MTT, to form insoluble purple formazan. An assay was performed as per the previously mentioned methodologies (Chaturvedi et al., 2018). The detailed methodology is described in Supplementary Method S2.

### Stimulation of cells

A total of 1 × 10^6^ cells/well were seeded in 6-well plates for 24 h. Following adherence, HPFBs were synchronized by incubating them with 2% DMEM for 24 h. For treatment with sildenafil and SB204741, two strategies were adopted. In the first strategy, i.e., the post-treatment strategy, HPFBs were initially treated with TGF-β1 (10 ng/mL) for 1 h and later incubated with TGF-β1 (10 ng/mL) and sildenafil or SB204741 (10 µM and 1 µM, respectively) for 24 h. In the second strategy, i.e., the pre-treatment strategy, HPFBs were pre-treated with sildenafil or SB204741 (10 µM and 1 µM, respectively) for 1 h and later stimulated with only TGF-β1 (10 ng/mL) for 24 h (Chaturvedi et al., 2018). In combination, similar post- and pre-treatment strategies were followed (Supplementary Figure S5). HPFBs obtained from the above two strategies were used for RNA extraction, and further quantitative real-time reverse transcriptase-polymerase chain reaction (qRT-PCR) was performed (Chaturvedi et al., 2018).

### RNA isolation and qRT-PCR

A total of 1 μg of RNA was processed for cDNA synthesis using a cDNA synthesis kit as per the manufacturer’s protocol. The qRT-PCR was performed in a LightCycler^®^ 480 System (Roche) using the 2X Maxima SYBR Green Master Mix (Roche) according to the manufacturer’s protocol. Primers for collagen type I alpha 1 chain (*COL1A1*), collagen type I alpha 2 chain (*COL1A2*), smooth muscle alpha (α)-2 actin (*ACTA2*), connective tissue growth factor (*CTGF*) and fibronectin 1 (*FN1*), and tissue inhibitor of metalloproteinase 1 (*TIMP1*), matrix metalloproteinase 2 (*MMP2*), transforming growth factor beta 1 (*TGF-Β1*), and glyceraldehyde 3-phosphate dehydrogenase (*GAPDH*) are listed in Supplementary Table S1. *GAPDH* was used as the internal standard (Chaturvedi et al., 2018). The detailed methodology is described in Supplementary Method S3.

### Enzyme-linked immunosorbent assay

The culture supernatant obtained after the stimulation of cells was analyzed for multiple pro- and anti-inflammatory cytokines. Cytokines such as IL-1β and IFN-γ were purchased from PeproTech, United States. Other cytokines such as IL-6, TNF-α, IL-10, IL-17, and IL-4 were purchased from R&D Systems, Minneapolis, United States. The TGF-β1 cytokine was purchased from Abcam, Boston, MA, United States. All these cytokines were measured by using an enzyme-linked immunosorbent assay (ELISA) as per the manufacturer’s protocol.

### Statistical analysis

Statistical analysis was performed with GraphPad Prism software (version 10.0, GraphPad Software, Boston, MA, USA). Data depicted in graphs represent the mean ± SEM for each group. The difference between the pharmacological treatments and non-pharmacological treatments was evaluated using the paired Student’s t-test. Multiple comparisons tests were performed through one-way ANOVA, followed by the Bonferroni *post hoc* test. A *p*-value of <0.05 was considered statistically significant.

## Results

### TGF-β1 stimulates the pro-fibrotic phenotype in HPFBs

qRT-PCR was performed on RNA isolated from HPFBs taken from PD patients and controls. HPFBs were stimulated at different doses of TGF-β1 (2–20 ng/mL). The percentage viability of HPFBs for TGF-β1 at 10 ng/mL was 80% (Supplementary Figure S2A).

TGF-β1 dose-dependently increased the pro-fibrotic gene mRNA expression with maximum induction at 10 ng/mL (Supplementary Figures S3A-C). Beyond 20 ng/mL, TGF-β1 did not further increase the expression of pro-fibrotic mRNA; it rather resulted in toxic effects (Supplementary Figures S3A-C).

### Serotonin regulates TGF-β1 levels in HPFBs

5-HT in increasing doses increased the mRNA levels of *TGF-Β1* on HPFBs obtained from both controls and PD patients (Supplementary Figure S1C).

### Pharmacological selective inhibition of type 5 of the PDE enzymes, 5-HT_2B_ receptor, and their combination exerts potent anti-fibrotic effects in an experimental model of PF

To observe whether selective inhibitors of isoform 5 of PDE enzymes, 5-HT_2B_ receptor, and their combination could suppress the TGF-β1-mediated fibrotic potential of HPFBs, the anti-fibrotic prospects of sildenafil, SB204741, and their combination were determined. The doses of sildenafil and SB204741 were selected based on the previously published data (Higuchi et al., 2015; Chaturvedi et al., 2018). HPFBs obtained from both controls and PD patients were incubated with sildenafil (10 µM) and SB204741 (1 µM), respectively, along with TGF-β1 at 10 ng/mL. The percentage viability for both the inhibitors at their respective selected doses was 80% individually as well as in combination (Supplementary Figures S2B-D, respectively). Of note, no evidence of the toxicity of anti-fibrotic doses of sildenafil (10 µM) and SB204741 (1 µM) was observed (Supplementary Figures S2B-D, respectively).

Furthermore, HPFBs obtained from both controls and PD patients were stimulated with TGF-β1 (Supplementary Figures S4A-F) and were incubated with sildenafil and SB204741 individually as well as in combination at increasing concentrations varying from 1 to 100 μM and 0.01 to 10 µM, respectively. There was a decrease in the mRNA levels of *COL1A1, COL1A2,* and *ACTA2* genes in a dose-dependent manner. The most prominent effects were observed at a concentration of 10 μM for sildenafil and 1 μM for SB204741. Hence, doses of 10 µM and 1 μM of sildenafil and SB204741 were chosen for further use.

To evaluate the pro-fibrotic pathway of TGF-β1, pro-fibrotic genes (*COL1A1, COL1A2, ACTA2, CTGF, FN1*, and *TGF-Β1*) responsible for HPFBs’ fibrotic potential were studied.

**FIGURE 1:**
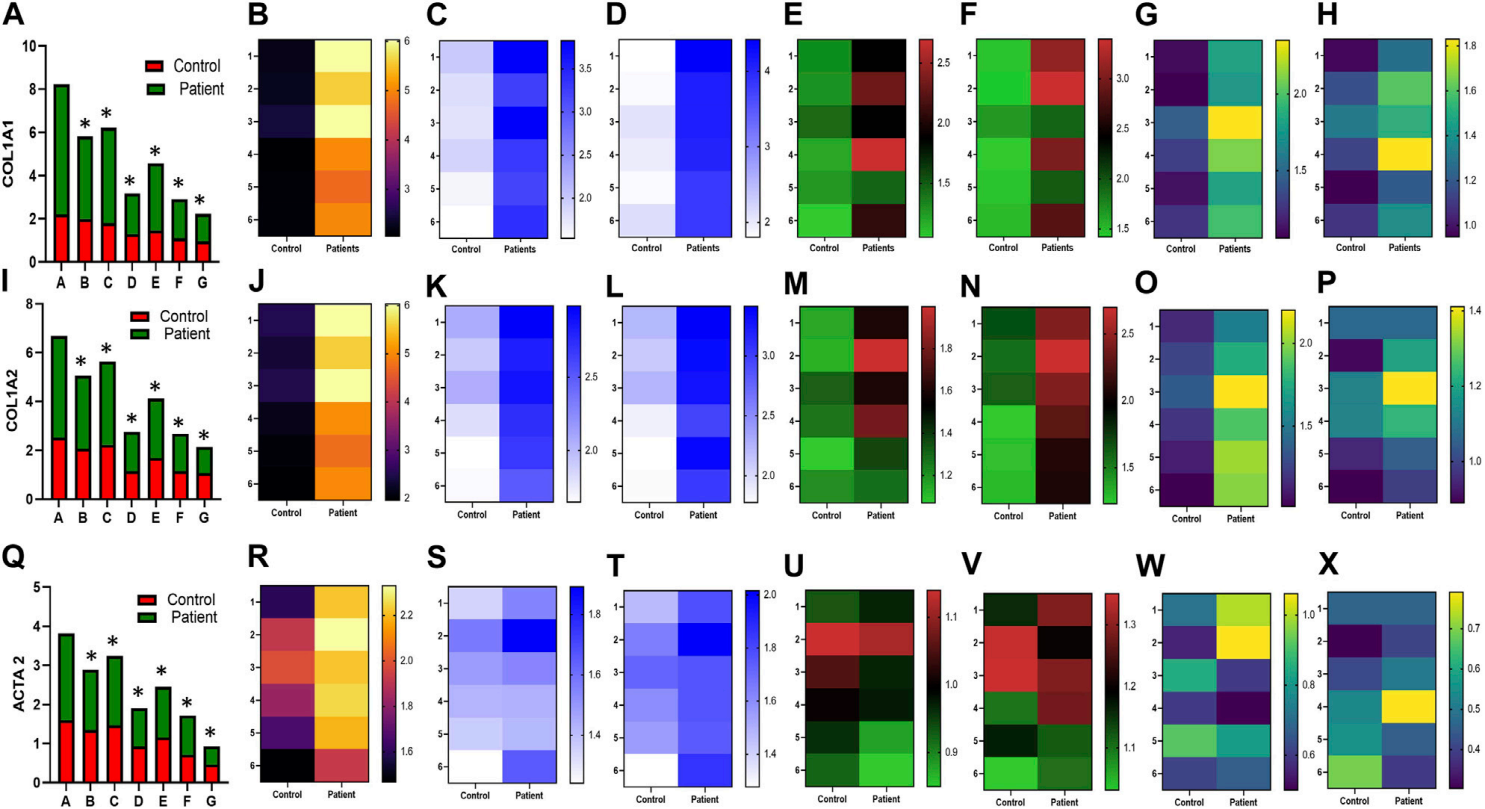
Effect of sildenafil, SB204741, and their combination on TGF-β1-induced pro-fibrotic *COL1A1*, *COL1A2,* and *ACTA2* gene mRNA expression in HPFBs obtained from an *in vitro* experimental model of PF: **(A)**, **(I)**, and **(Q)** represent the fold change in the mRNA levels of *COL1A1, COL1A2,* and *ACTA2* genes, respectively, in HPFBs obtained from controls and PD patients cultured with only TGF-β1 (bar 1). HPFBs were pre-treated with TGF-β1 (10 ng/mL) for 1 h followed by co-culture with TGF-β1 (10 ng/mL) and a (10 μM) dose of sildenafil (bar 2) and a (1 μM) dose of SB204741 (bar 3), respectively, for 24 h. Fibroblasts were pre-treated with a (10 μM) dose of sildenafil (bar 4) and a (1 μM) dose of SB204741 (bar 5), respectively, for 1 h and later incubated with TGF-β1 (10 ng/mL) only for 24 h. Bar 6 depicts the post-treatment strategy with a (10 μM) dose of sildenafil and a (1 μM) dose of SB204741 in combination, and the pre-treatment strategy with a (10 μM) dose of sildenafil and a (1 μM) dose of SB204741 in combination is depicted by bar 7. **(B-H, J-P, R-X)** Heat map showing the significant gene expression changes for *COL1A1, COL1A2, and ACTA2* involved in peritoneal fibrosis. The mRNA levels of *COL1A1, COL1A2, and ACTA2* after the above treatment strategies were determined using the 2^−ΔΔCT^ method. Data were normalized with *GAPDH* as the housekeeping gene. The combination of sildenafil and SB204741 decreased mRNA levels to a greater extent than either drug alone. The experiment was performed in three independent series. RT-PCR analysis of the expression of *COL1A1, COL1A2, and ACTA2* in HPFBs cultured in the presence of sildenafil, SB204741, and their combination compared with HPFBs cultured in the presence of TGF-β1 (mean ± SEM; *p ≤ 0.05 by paired Student’s t-test; ns = non-significant).

**FIGURE 2:**
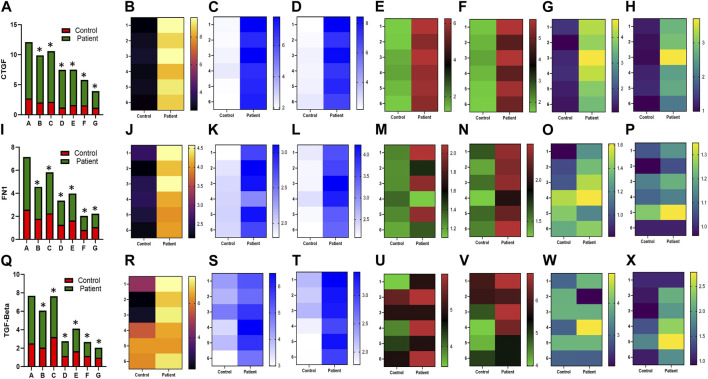
Effect of sildenafil, SB204741, and their combination on TGF-β1-induced pro-fibrotic *CTGF, FN1*, and *TGF-Β1* gene mRNA expression in HPFBs obtained from the *in vitro* experimental model of PF: **(A)**, **(I)** and **(Q)** represent the fold change in mRNA levels of *CTGF, FN1*, and *TGF-Β1* genes in HPFBs obtained from controls and PD patients cultured with only TGF-β1 (bar 1). HPFBs were pre-treated with TGF-β1 (10 ng/mL) for 1 h followed by co-culture with TGF-β1 (10 ng/mL) and a (10 μM) dose of sildenafil (bar 2) and a (1 μM) dose of SB204741 (bar 3), respectively, for 24 h. Fibroblasts were pre-treated with a (10 μM) dose of sildenafil (bar 4) and a (1 μM) dose of SB204741 (bar 5) for 1 h and later incubated with TGF-β1 (10 ng/mL) only for 24 h. Bar 6 depicts the post-treatment strategy with a (10 μM) dose of sildenafil and a (1 μM) dose of SB204741 in combination, and the pre-treatment strategy with a (10 μM) dose of sildenafil and a (1 μM) dose of SB204741 in combination is depicted by bar 7. **(B-H, J-P, R-X)** Heat map showing the significant gene expression changes for *CTGF, FN1*, and *TGF-Β1* involved in peritoneal fibrosis. mRNA levels of *CTGF, FN1*, and *TGF-Β1* after above treatment strategies were determined using the 2^−ΔΔCT^ method. Data were normalized with *GAPDH* as the housekeeping gene. The combination of sildenafil and SB204741 decreased mRNA levels to a greater extent than either drug alone. The experiment was performed in three independent series. RT-PCR analysis of the expression of *CTGF, FN1*, and *TGF-Β1* in HPFBs cultured in the presence of sildenafil, SB204741, and their combination compared with HPFBs cultured in the presence of TGF-β1 (mean ± SEM; *p ≤ 0.05 by paired Student’s t-test; ns = non-significant).

HPFBs were synchronized with DMEM containing 2% FBS for 24 h. Subsequently, HPFBs obtained from both controls and PD patients were stimulated with TGF-β1 at 10 ng/mL, which upregulated the levels of the *COL1A1* (Figures 1A-H)*, COL1A2* (Figures 1I-P)*, ACTA2* (Figures 1Q-X)*, CTGF* (Figures 2A-H), *FN1* (Figures 2I-P), and *TGF-Β1* (Figures 2Q-X) mRNA at 24 h.

In the post-treatment strategy, mRNA levels of *COL1A1* (Figures 1A-H)*, COL1A2* (Figures 1I-P)*, ACTA2* (Figures 1Q-X)*, CTGF* (Figures 2A-H), *FN1* (Figures 2I-P), and *TGF-Β1* (Figures 2Q-X) were significantly downregulated by sildenafil and SB204741 individually as well as in combination compared to stimulation with TGF-β1 in HPFBs.

**FIGURE 3:**
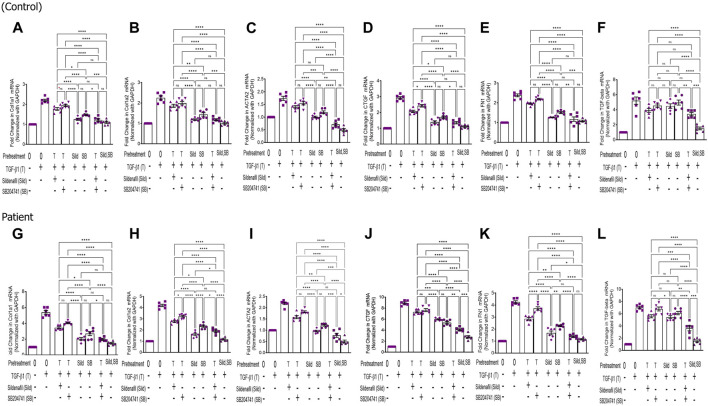
Effect of sildenafil and SB204741 and their combination on TGF-β1-induced pro-fibrotic gene mRNA expression in HPFBs obtained from the *in vitro* experimental model of PF: (A–L) HPFBs obtained from **(A–F)** controls and (**G–L)** PD patients were cultured with only media (bar 1) and with TGF-β1 (bar 2). HPFBs were pre-treated with TGF-β1 (10 ng/mL) for 1 h followed by co-culture with TGF-β1 (10 ng/mL) and a (10 μM) dose of sildenafil (bar 3) and a (1 μM) dose of SB204741 (bar 4) for 24 h. Fibroblasts were pre-treated with a (10 μM) dose of sildenafil (bar 5) and a (1 μM) dose of SB204741 (bar 6) for 1 h and later incubated with TGF-β1 (10 ng/mL) only for 24 h. The post-treatment strategy with a (10 μM) dose of sildenafil and a (1 μM) dose of SB204741 in combination is depicted by bar 7, and the post-treatment strategy with a (10 μM) dose of sildenafil and a (1 μM) dose of SB204741 in combination is depicted by bar 8. mRNA levels of *COL1A1* **(A,G)**, *COL1A2* **(B,H)**, *ACTA2* **(C,I)**, *CTGF* **(D,J)**, *FN1* **(E,K)**, and *TGF-Β1* **(F,L)** after above treatment strategies were determined using the 2^−ΔΔCT^ method. Data were normalized with *GAPDH* as the housekeeping gene. The combination of sildenafil and SB204741 decreased mRNA levels to a greater extent than either drug alone. The experiment was performed in three independent series. Data are expressed as mean ± SEM of n = 3 independent experiments. Significance was determined by one-way ANOVA. *, *p* ≤ 0.05; **, *p* ≤ 0.01 was considered as statistically significant; ns = non-significant.

In the pre-treatment strategy, compared to TGF-β1-stimulated HPFBs, treatment of both the inhibitors individually as well as in combination significantly downregulated mRNA levels of *COL1A1* (Figures 1A-H)*, COL1A2* (Figures 1I-P)*, ACTA2* (Figures 1Q-X)*, CTGF* (Figures 2A-H), *FN1* (Figures 2I-P), and *TGF-Β1* (Figures 2Q-X). In the pre-treatment strategy, the combination of sildenafil and SB204741 almost completely abrogated the potential of *ACTA2*. The anti-fibrotic effect of the combination of sildenafil and SB204741 was higher than that of each drug alone. Multiple comparisons between the single pharmacological treatment and the combination pharmacological treatment in HPFBs on mRNA levels of pro-fibrotic genes are depicted in Figures 3A-L for controls and patients, respectively [*COL1A1* (Figures 3A, G)*, COL1A2* (Figures 3B, H)*, ACTA2* (Figures 3C, I)*, CTGF* (Figures 3D, J), *FN1* (Figures 3E, K), and *TGF-Β1* (Figures 3F, L)].

**FIGURE 4:**
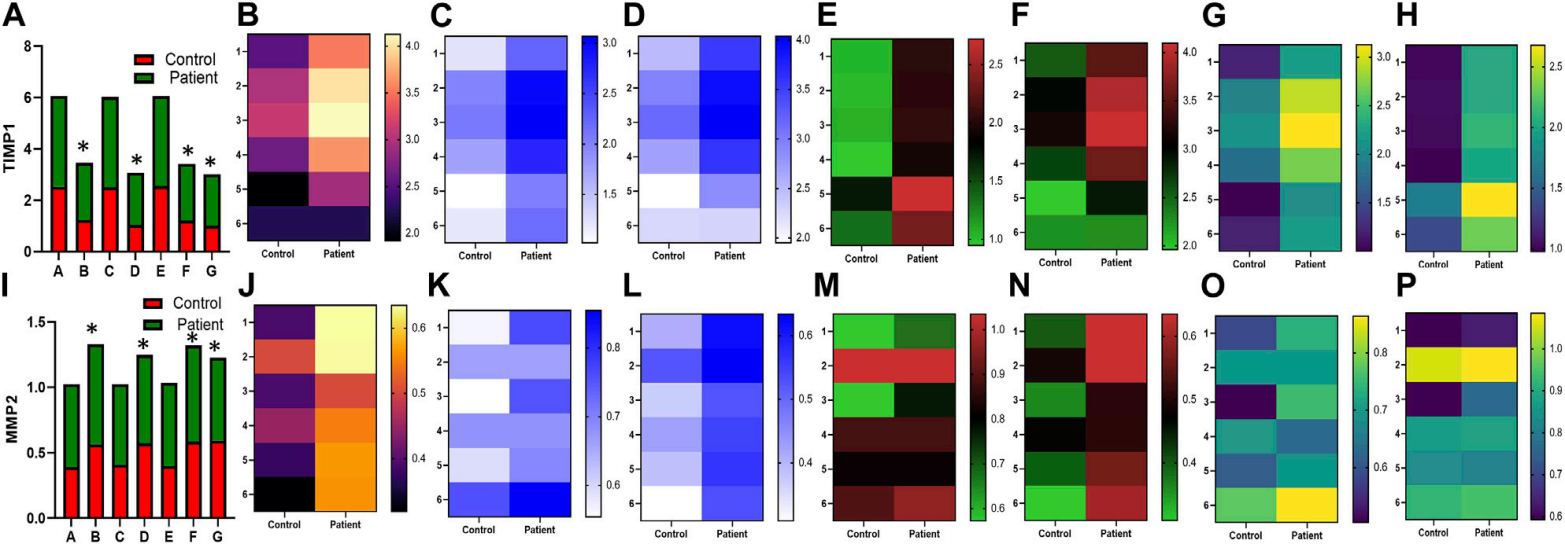
Effect of sildenafil, SB204741, and their combination on TGF-β1-induced anti-fibrotic gene mRNA expression in HPFBs obtained from an *in vitro* experimental model of PF: **(A)** and **(I)** represent fold change in mRNA levels of *TIMP1* and *MMP2* genes, respectively, in HPFBs obtained from controls and PD patients cultured with only TGF-β1 (bar 1). HPFBs were pre-treated with TGF-β1 (10 ng/mL) for 1 h followed by co-culture with TGF-β1 (10 ng/mL) and a (10 μM) dose of sildenafil (bar 2) and a (1 μM) dose of SB204741 (bar 3) for 24 h. Fibroblasts were pre-treated with a (10 μM) dose of sildenafil (bar 4) and a (1 μM) dose of SB204741 (bar 5) for 1 h and later incubated with TGF-β1 (10 ng/mL) only for 24 h. Bar 6 depicts the post-treatment strategy with a (10 μM) dose of sildenafil and a (1 μM) dose of SB204741 in combination, and the pre-treatment strategy with a (10 μM) dose of sildenafil and a (1 μM) dose of SB204741 in combination is depicted by bar 7. **(B-H, J-P)** Heat map showing the significant gene expression changes for *TIMP1* and *MMP2* involved in peritoneal fibrosis. mRNA levels of *TIMP1* and *MMP2* after above treatment strategies were determined using the 2^−ΔΔCT^ method. Data were normalized with *GAPDH* as the housekeeping gene. The experiment was performed in three independent series. RT-PCR analysis of the expression of *TIMP1* and *MMP2* in HPFBs cultured in the presence of sildenafil, SB204741, and their combination compared with HPFBs cultured in the presence of TGF-β1 (mean ± SEM; *p ≤ 0.05; **, *p* ≤ 0.01 by paired Student’s t-test; ns = non-significant).

**FIGURE 5:**
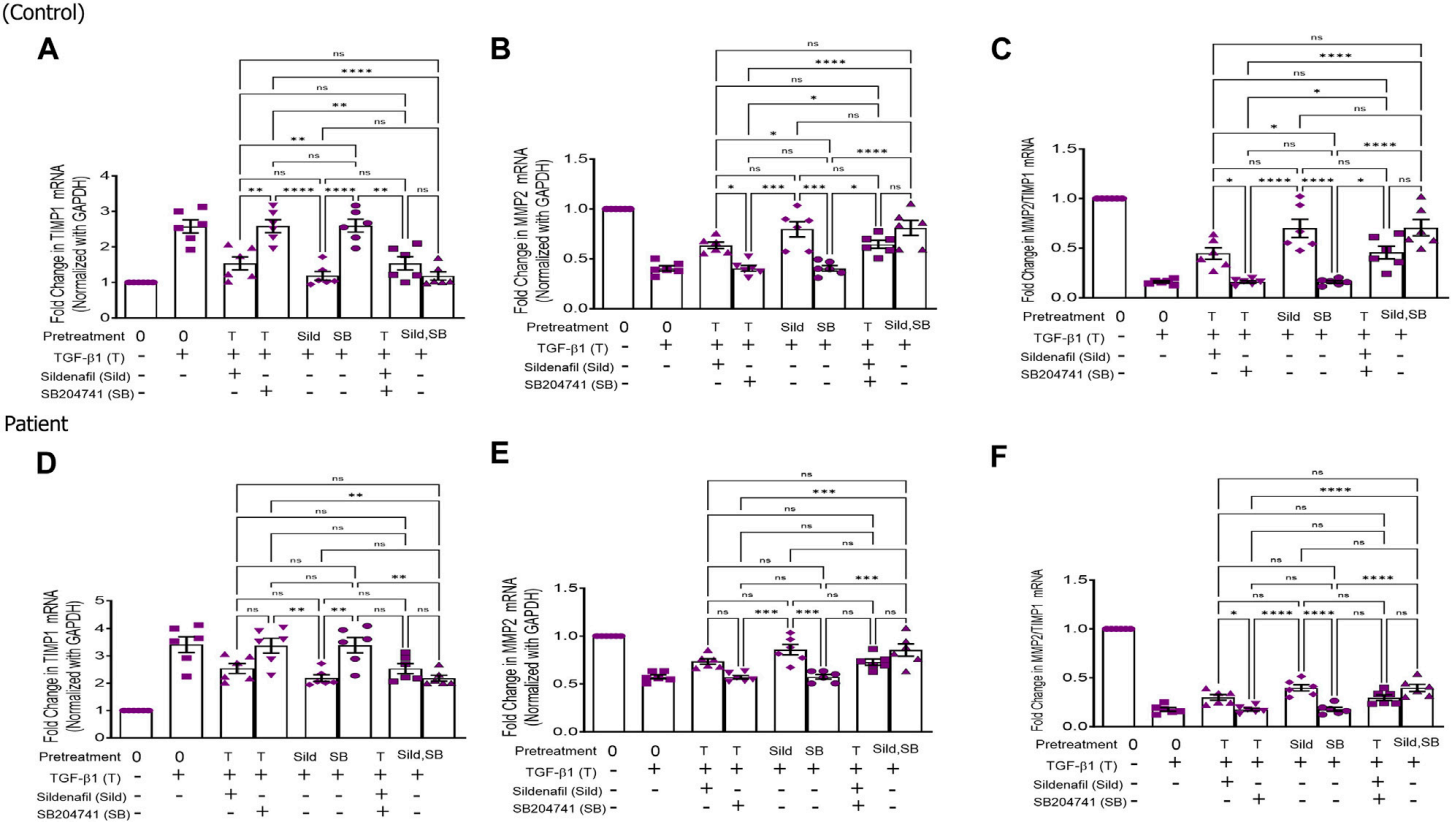
Effect of sildenafil, SB204741, and their combination on TGF-β1-induced anti-fibrotic gene mRNA expression in HPFBs obtained from an *in vitro* experimental model of PF: (A–F) HPFBs obtained from **(A–C)** controls and **(D–F)** PD patients were cultured with only media (bar 1) and with TGF-β1 (A–C, controls; D–F, PD patients, bar 2). HPFBs were pre-treated with TGF-β1 (10 ng/mL) for 1 h followed by co-culture with TGF-β1 (10 ng/mL) and a (10 μM) dose of sildenafil (bar 3) and a (1 μM) dose of SB204741 (bar 4) for 24 h. Fibroblasts were pre-treated with a (10 μM) dose of sildenafil (bar 5) and a (1 μM) dose of SB204741 (bar 6) for 1 h and later incubated with TGF-β1 (10 ng/mL) only for 24 h. The post-treatment strategy with a (10 μM) dose of sildenafil and a (1 μM) dose of SB204741 in combination is depicted by bar 7, and the post-treatment strategy with a (10 μM) dose of sildenafil and a (1 μM) dose of SB204741 in combination is depicted by bar 8. mRNA levels of *TIMP1* **(A,D)**, *MMP2* **(B,E)**, and *MMP2*/*TIMP1* **(C,F)** after above treatment strategies were determined using the 2^−ΔΔCT^ method. Data were normalized with *GAPDH* as the housekeeping gene. The combination of sildenafil and SB204741 increased the MMP2/TIMP1 ratio to a greater extent than either drug alone. The experiment was performed in three independent series. Data are expressed as mean ± SEM of n = 3 independent experiments. Significance was determined by one-way ANOVA. *, *p* ≤ 0.05; **, *p* ≤ 0.01 was considered statistically significant; ns = non-significant.

The anti-fibrotic gene, *MMP2*, and its inhibitor *TIMP1* were evaluated in HPFBs obtained from both controls and PD patients. *TIMP1* expression was upregulated in HPFBs upon incubation with TGF-β1, which decreased on treatment with sildenafil individually. However, *TIMP1* expression did not have any effect on treatment with SB204741 independently. In combination, *TIMP1* expression decreased in comparison to the expression obtained upon TGF-β1 stimulation (Figures 4A-H). *MMP2* expression on TGF-β1 incubation reduced in HPFBs obtained from both controls and PD patients. However, sildenafil reversed and increased *MMP2* expression. SB204741 treatment did not have any effect on *MMP2* expression. In combination, *MMP2* expression increased in HPFBs obtained from both controls and PD patients in comparison to TGF-β1 stimulation (Figures 4I-P). Multiple comparisons between the single pharmacological treatment and the combination pharmacological treatment in HPFBs on mRNA levels of anti-fibrotic genes are depicted in Figures 5A-F for controls and patients, respectively [*TIMP1* (Figures 5A, D)*, MMP2* (Figures 5B, E)]. MMP activity is regulated by TIMPs, which bind to MMPs in a 1:1 stoichiometric ratio (Deardorff and spinale, 2009). Overall, the ratio between *MMP2* and *TIMP1* signifies the efficacy of an anti-fibrotic response. Due to treatment with TGFβ1, this ratio was significantly reduced compared to that of the blank. However, treatment with sildenafil individually as well as in combination with SB204741 restored this ratio, but no response was observed on the ratio on treatment with SB204741 individually (Figures 5C, F).

### Effect of sildenafil, SB204741, and their combination on TGF-β1-induced pro- and anti-inflammatory cytokines production in HPFBs

**FIGURE 6:**
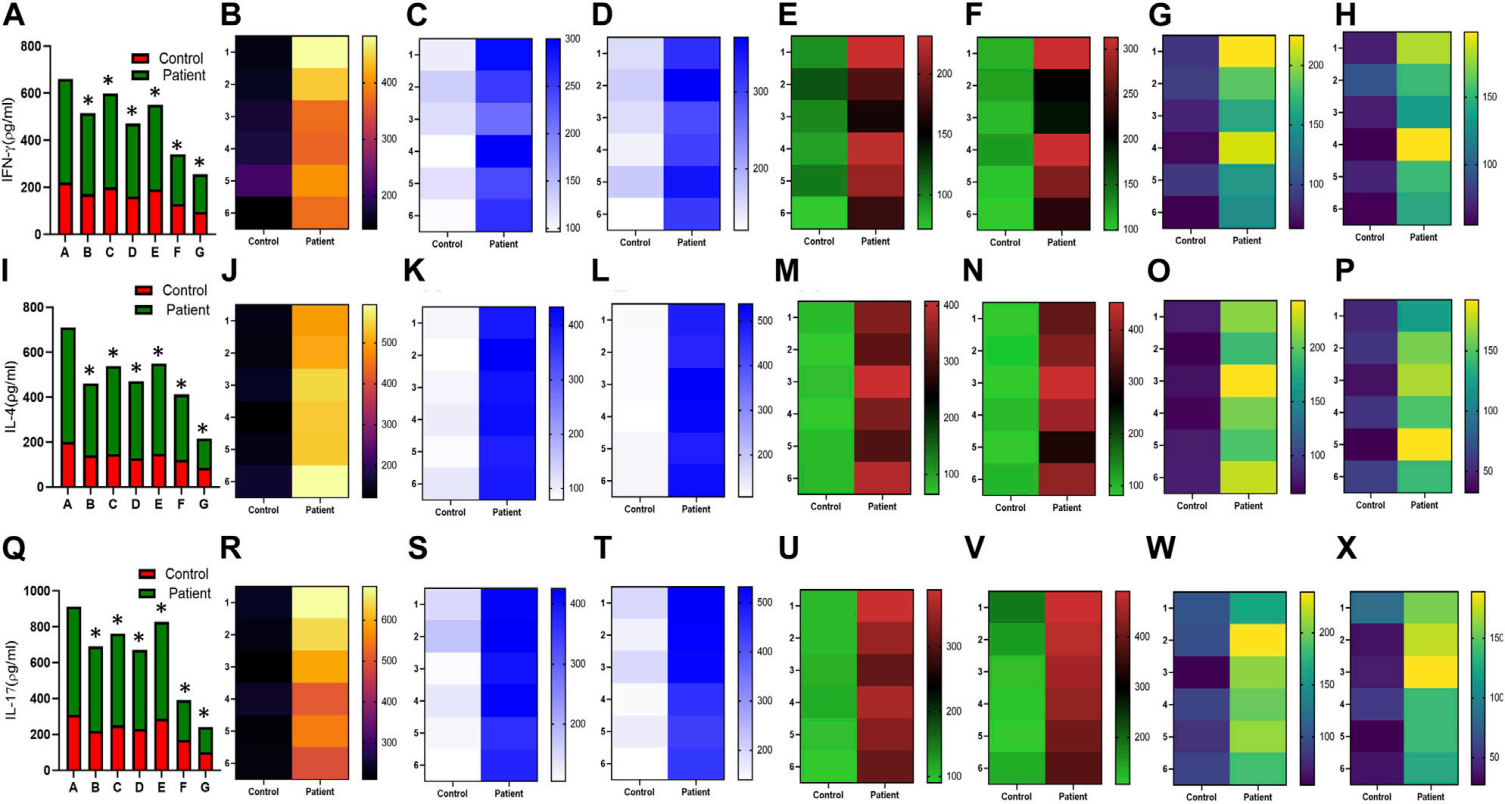
Effect of sildenafil, SB204741, and their combination on TGF-β1-induced pro inflammatory cytokines IFN-γ, IL-4, and IL-17 in HPFBs obtained from an *in vitro* experimental model of PF: Heat map showing the significant changes in the levels of IFN-γ, IL-4, and IL-17 cytokines involved in peritoneal fibrosis.**(A)**, **(I)**, and **(Q)** represent the histogram depicting the change in levels of proinflammatory IFN-γ, IL-4, and IL-17 cytokines in HPFBs obtained from controls and PD patients cultured with only TGF-β1 (bar 1). HPFBs were pre-treated with TGF-β1 (10 ng/mL) for 1 h followed by co-culture with TGF-β1 (10 ng/mL) and a (10 μM) dose of sildenafil (bar 2) and a (1 μM) dose of SB204741 (bar 3) for 24 h. Fibroblasts were pre-treated with a (10 μM) dose of sildenafil (bar 4) and a (1 μM) dose of SB204741 (bar 5) for 1 h and later incubated with TGF-β1 (10 ng/mL) only for 24 h. Bar 6 depicts the post-treatment strategy with a (10 μM) dose of sildenafil and a (1 μM) dose of SB204741 in combination, and the pre-treatment strategy with a (10 μM) dose of sildenafil and a (1 μM) dose of SB204741 in combination is depicted by bar 7. **(B-H, J-P, R-X)** Heat map showing the significant cytokine profile changes for IFN-γ, IL-4, and IL-17 involved in peritoneal fibrosis. The combination of sildenafil and SB204741 decreased IFN-γ, IL-4, and IL-17 levels to a greater extent than either drug alone. The experiment was performed in three independent series (mean ± SEM; *p ≤ 0.05 by paired Student’s t-test; ns = non-significant).

**FIGURE 7:**
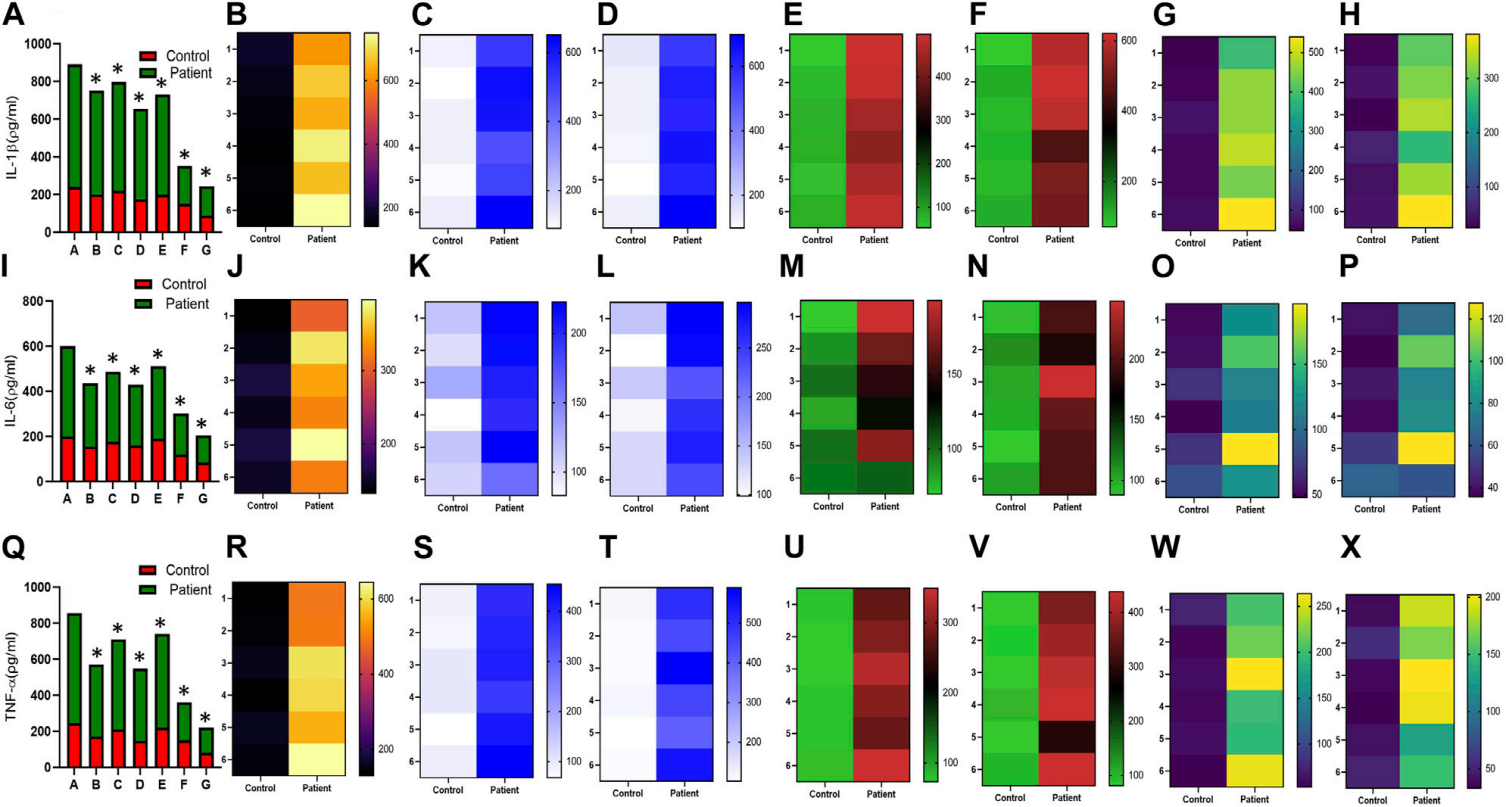
Effect of sildenafil, SB204741, and their combination on TGF-β1-induced pro inflammatory cytokines IL-1β, IL-6, and TNF-α in HPFBs obtained from an *in vitro* experimental model of PF: Heat map showing the significant changes in the levels of IL-1β, IL-6, and TNF-α cytokines involved in peritoneal fibrosis.**(A)**, **(I)**, and **(Q)** represent the histogram depicting the change in levels of proinflammatory IL-1β, IL-6, and TNF-α cytokines in HPFBs obtained from controls and PD patients cultured with only TGF-β1 (bar 1). HPFBs were pre-treated with TGF-β1 (10 ng/mL) for 1 h followed by co-culture with TGF-β1 (10 ng/mL) and a (10 μM) dose of sildenafil (bar 2) and a (1 μM) dose of SB204741 (bar 3) for 24 h. Fibroblasts were pre-treated with a (10 μM) dose of sildenafil (bar 4) and a (1 μM) dose of SB204741 (bar 5) for 1 h and later incubated with TGF-β1 (10 ng/mL) only for 24 h. Bar 6 depicts the post-treatment strategy with a (10 μM) dose of sildenafil and a (1 μM) dose of SB204741 in combination, and the pre-treatment strategy with a (10 μM) dose of sildenafil and a (1 μM) dose of SB204741 in combination is depicted by bar 7. **(B-H, J-P, R-X)** Heat map showing the significant cytokine profile changes for IL-1β, IL-6, and TNF-α involved in peritoneal fibrosis. The combination of sildenafil and SB204741 decreases IL-1β, IL-6, and TNF-α levels to a greater extent than either drug alone. The experiment was performed in three independent series (mean ± SEM; *p ≤ 0.05 by paired Student’s t-test; ns = non-significant).

**FIGURE 8:**
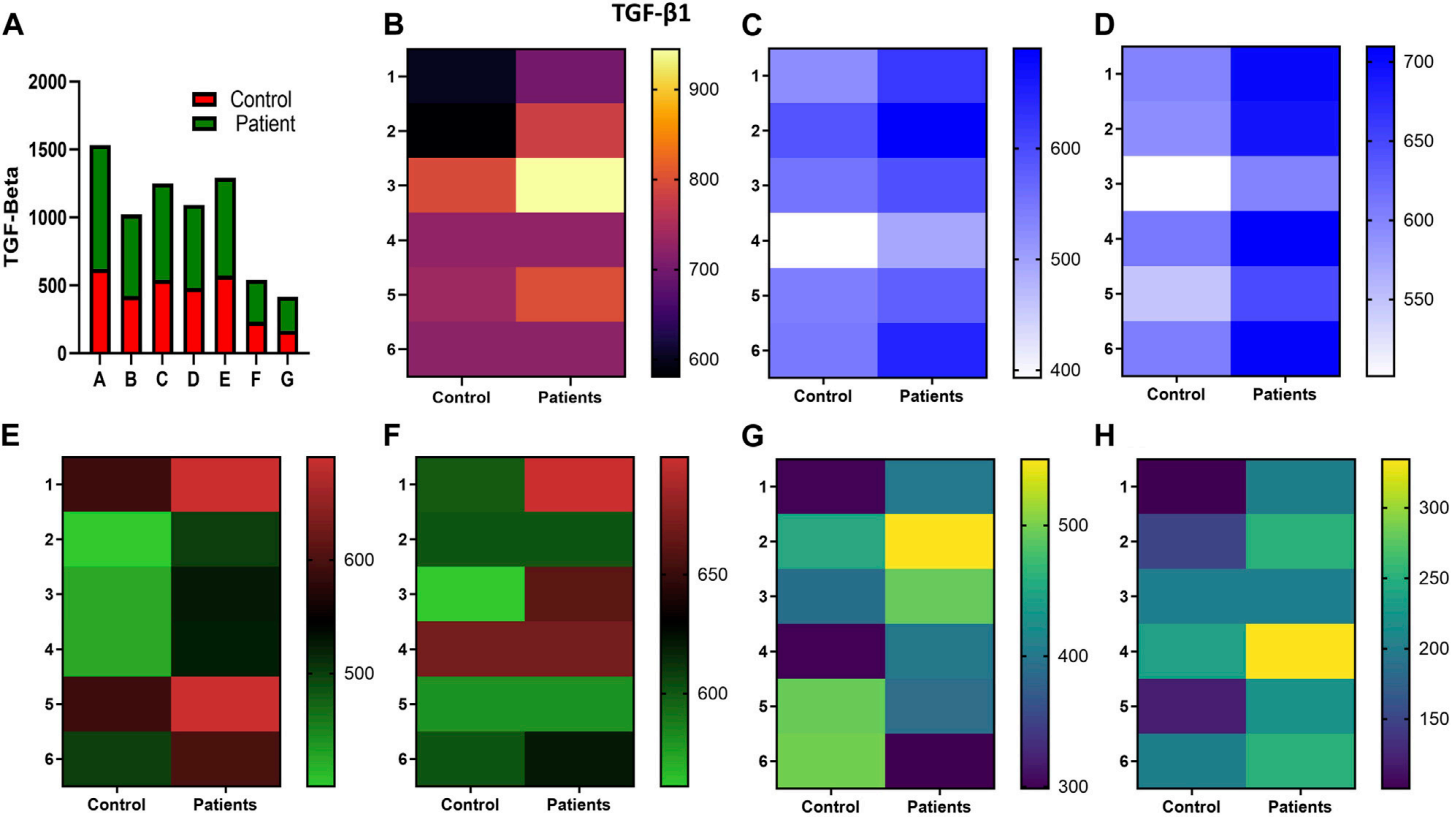
Effect of sildenafil, SB204741, and their combination on TGF-β1-induced anti-inflammatory cytokine TGF-β1 in HPFBs obtained from an *in vitro* experimental model of PF: Heat map showing the significant changes in the levels of TGF-β1 cytokine involved in peritoneal fibrosis. **(A)** represents the histogram depicting the change in levels of proinflammatory TGF-β1 cytokine in HPFBs obtained from controls and PD patients cultured with only TGF-β1 (bar 1). HPFBs were pre-treated with TGF-β1 (10 ng/mL) for 1 h followed by co-culture with TGF-β1 (10 ng/mL) and a (10 μM) dose of sildenafil (bar 2) and a (1 μM) dose of SB204741 (bar 3) for 24 h. Fibroblasts were pre-treated with a (10 μM) dose of sildenafil (bar 4) and a (1 μM) dose of SB204741 (bar 5) for 1 h and later incubated with TGF-β1 (10 ng/mL) only for 24 h. Bar 6 depicts the post-treatment strategy with a (10 μM) dose of sildenafil and a (1 μM) dose of SB204741 in combination, and the pre-treatment strategy with a (10 μM) dose of sildenafil and a (1 μM) dose of SB204741 in combination is depicted by bar 7. **(B–H)** Heat map showing the significant cytokine profile changes for TGF-β1 involved in peritoneal fibrosis. The combination of sildenafil and SB204741 decreased TGF-β1 cytokine levels to a greater extent than either drug alone. The experiment was performed in three independent series (mean ± SEM; *p ≤ 0.05 by paired Student’s t-test; ns = non-significant).

**FIGURE 9:**
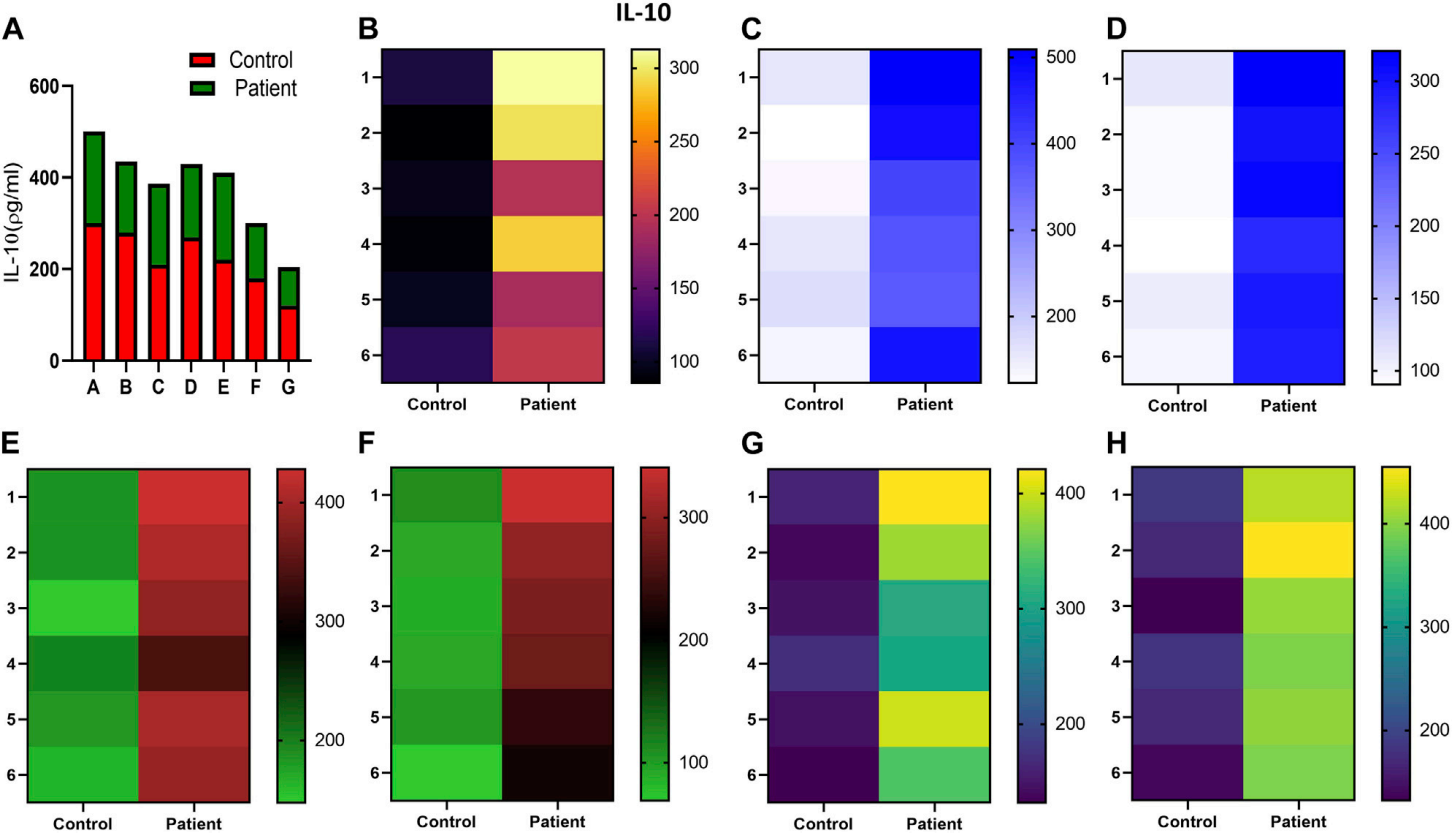
Effect of sildenafil, SB204741, and their combination on TGF-β1-induced anti-inflammatory cytokine IL-10 in HPFBs obtained from an *in vitro* experimental model of PF: Heat map showing the significant changes in the levels of IL-10 cytokine involved in peritoneal fibrosis. **(A)** represents the histogram depicting the change in levels of anti-inflammatory IL-10 cytokine in HPFBs obtained from controls and PD patients cultured with only TGF-β1 (bar 1). HPFBs were pre-treated with TGF-β1 (10 ng/mL) for 1 h followed by co-culture with TGF-β1 (10 ng/mL) and a (10 μM) dose of sildenafil (bar 2) and a (1 μM) dose of SB204741 (bar 3) for 24 h. Fibroblasts were pre-treated with a (10 μM) dose of sildenafil (bar 4) and a (1 μM) dose of SB204741 (bar 5) for 1 h and later incubated with TGF-β1 (10 ng/mL) only for 24 h. Bar 6 depicts the post-treatment strategy with a (10 μM) dose of sildenafil and a (1 μM) dose of SB204741 in combination, and the pre-treatment strategy with a (10 μM) dose of sildenafil and a (1 μM) dose of SB204741 in combination is depicted by bar 7. **(B–H)** Heat map showing the significant cytokine profile changes for IL-10 involved in peritoneal fibrosis. The experiment was performed in three independent series (mean ± SEM; *p ≤ 0.05 by paired Student’s t-test; ns = non-significant).

Increased expression of proinflammatory cytokines in the sub-mesothelial zone was considered a characteristic pathological change in the fibrotic PM (Lai et al., 2007). To demonstrate, we examined the levels of proinflammatory cytokines IFN-γ IL-4, IL-17, IL-1β, IL-6, TNF-α, and TGFβ1 and anti-inflammatory cytokine IL-10 in the culture supernatant of HPFBs obtained from both controls and PD patients. Stimulation of HPFBs with TGF-β1 led to an increase in the levels of the IFN-γ (Figures 6A-H), IL-4 (Figures 6I-P), IL-17 (Figures 6Q-X), IL-1β (Figures 7A-H), IL-6 (Figures 7I-P), TNF-α (Figures 7Q-X), and TGF-β1 (Figures 8A-H) in the PD patient group compared to controls. In this study, treatment of HPFBs with sildenafil (Laxmi et al., 2019) and SB204741 individually as well as in combination inhibited TGF-β1-induced proinflammatory cytokine production. Furthermore, IL-10 levels on TGF-β1 incubation increased in both PD patients and controls. Sildenafil treatment increased the levels of IL-10 compared to TGF-β1 stimulation of HPFBs. SB204741 alone did not affect the production of IL-10 in the culture supernatant. In combination, IL-10 levels were increased in both PD patients and controls in comparison to TGF-β1 stimulation (Figures 9A-H).

## Discussion

Studies in the past documented that the EMT process (*ACTA2* gene plays a critical role) of PMCs may be a potential target for therapeutic intervention to preserve the morphology and functions of PM in PD patients (Margetts and Bonniaud, 2003; Aroeira et al., 2007; Devuyst et al., 2010). Recent studies showed that during PD, tissue injury caused by the non-physiological peritoneal dialysis solution upregulates a broad range of genes, cytokines, and other factors (Aroeira et al., 2007; Devuyst et al., 2010). Among them, TGF-β1 acts as a crucial factor responsible for non-physiological peritoneal dialysis solution-induced worsening of the PM (Yao et al., 2008; Patel et al., 2010; Zhu et al., 2010).

**FIGURE 10:**
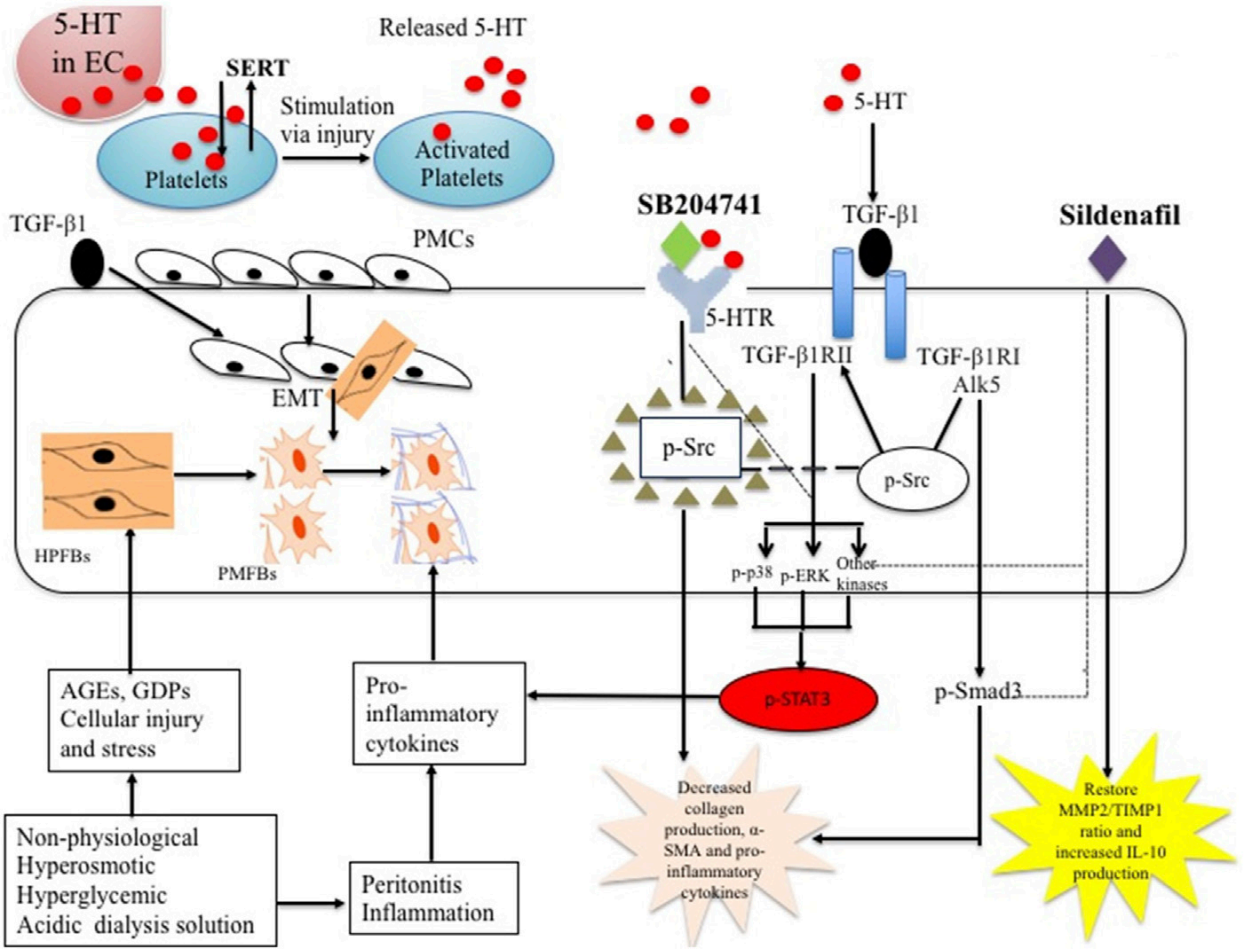
Proposed scheme of mechanism of combination use of sildenafil and SB204741 in ameliorating fibrotic potential of human peritoneal fibroblasts: The ubiquitous nature of PF occurs in PD patients in response to a variety of injurious agents to which the peritoneum is routinely exposed. The most important agents responsible include components of non-physiological dialysis fluid, mainly glucose, GDPs, and AGEs that can be linked to PF. Long-term continuous exposure to the non-physiological dialysis fluid results in a reduction in UF, loss of the mesothelial cell layer, and sub-mesothelial layer thickening that constitute increased PMFBs, collagen deposition, and sub-mesothelial fibrosis. Pathologically, during the EMT process of PMCs, HPFBs invade the sub-mesothelial compact zone, leading to loss of the mesothelial cell layer, differentiation of fibroblasts to collagen-producing PMFBs, excessive deposition of the extracellular matrix (ECM) that disrupts normal peritoneal architecture, and homeostasis and further leads to progressive fibrosis of the sub-mesothelial region. In response to long-term PD, PMFBs may originate from resident HPFBs and also from the mesothelial layer of PM via EMT. TGF-β1 induces the EMT process of PMCs and PMFBs with excessive collagen production, which are maintained through production of proinflammatory cytokines. Released 5-HT from EC can be transported into neurons and platelets through SERT, most of which is stored in the dense granules of the platelets and transported to the peripheral blood. Platelets, upon activation through stimulation via vascular injury or pathogen, release 5-HT to a wide number of tissues and, importantly, will accumulate within injured tissues, releasing 5-HT upon cellular stress and injury. 5-HT converts latent TGF-β1 to active TGF-β1. The TGF-β1 ligand first binds to the homodimer of TGF-βRII that provides the activating signal to TGF-βRI, which leads to phosphorylation (p) of Smad3 and mediates canonical signaling. On the other hand, these now activated TGF-β1 phosphorylate (p) Src located in the cytosol (located very near to TGF-βRI, indicated by circle), which, once phosphorylated, move toward TGF-βRII and leads to its activation at a different site and causes activation of various MAP kinases that all culminate into phosphorylation of STAT3. Treatment with SB204741 physically sequesters p-Src (indicated by square circled by small green triangles), preventing non-canonical TGF-β1 signaling by restricting the Src’s ability to phosphorylate TGF-βRII that inhibits various p-ERK and MAP kinases (indicated by ---- line), ultimately leading to inhibition of p-STAT3 (indicated by the red circle), which leads to decreased collagen, α-SMA, and proinflammatory cytokines. Sildenafil also inhibits non-canonical and canonical TGF-β1 signaling (indicated by ---- line) and, subsequently, restores the MMP2/TIMP1 ratio and IL-10 levels as well as decreased collagen, α-SMA, and proinflammatory cytokines. PD: continuous ambulatory peritoneal dialysis; GDPs: glucose degradation products; AGEs: advanced glycation end-products; UF: ultrafiltration; HPFBs: human peritoneal fibroblasts; EMT: epithelial-to-mesenchymal transition; PMFBs: peritoneal myo-fibroblasts; α-SMA: alpha smooth muscle actin; PMCs: peritoneal mesothelial cells; 5-HT: serotonin; TGF-β1: transforming growth factor beta 1; p-Smad3: phosphorylated Smad3; p-Src: phosphorylated Src; p-ERK: phosphorylated extracellular signal regulated kinases; p-p38: phosphorylated p38; p-STAT3: phosphorylated signal transducer and activator of transcription 3; TGF-βRI: type I receptor of TGF-β1; TGF-βRII: type II receptor of TGF-β1; MMP2: matrix metalloproteinase 2; TIMP1: tissue inhibitor of metalloproteinase.

In this study, we have demonstrated the anti-fibrotic potential of sildenafil, SB204741, and their combination via their *in vitro* incubation in TGF-β1-stimulated HPFBs, suggesting the involvement of 5-HT/TGF β1 signaling in PF as well as the molecular mechanisms involved in the process (Figure 10). Vasculopathy in fibrosis leads to endothelial injury, and subsequent platelet activation releases 5-HT, which converts latent TGF-β1 in plasma to active TGF-β1 (El-Tanbouly et al., 2017). It is worth commenting that PMFBs have been reported to be marked in peritoneal biopsies from PD patients (Jiménez-Heffernan et al., 2004). Therefore, we hypothesized using the combination of sildenafil and SB204741, which may prove to be a beneficial strategy in TGF-β1-induced activation of HPFBs isolated from PD patients and controls.

At the molecular level, the basic etiology for fibrosis is supposed to be initiated by activation from quiescent HPFBs to contractile PMFBs, which are characterized by the increased ECM synthesis and expression of α-SMA, leading to fibrotic changes in the PM (Loureiro et al., 2011). The major impacts of our study are: 1) combination treatment in comparison to individual treatments proved more beneficial in inhibiting the fibrotic potential of HPFBs and significantly decreased it; 2) combination treatment almost completely mitigated *ACTA2* and, thus, might arrest 5-HT/TGF-β1-mediated PMFB activation from resident HPFBs; 3) combination treatment decreased proinflammatory cytokine levels and increased anti-inflammatory cytokine IL-10 levels. Additionally, in the above process, MMPs are downregulated and TIMPs are upregulated. Therefore, to eliminate the probability that induction of matrix-degrading enzymes or their inhibitors suppress the decreased synthesis of the ECM, we observed the expression of MMPs and TIMPs in HPFBs incubated with TGF-β1, sildenafil, and SB204741. However, SB204741 did not affect the expression of the *MMP2/TIMP1* ratio, but surprisingly, sildenafil alone, as well as in combination, restored the expression of *MMP2/TIMP1*, an observation which was not reported till date.

SB204741 has been reported to decrease α-SMA expression in AVICs and dermal fibroblasts incubated with TGF-β1 via physical sequestration of phosphorylated-Src (p-Src) (Hutcheson et al., 2012; Chaturvedi et al., 2018). The above physical sequestration of p-Src results in an inhibition of phosphorylation of p-38, which is necessary for myofibroblast differentiation and leads to a decrease in α-SMA expression (Hutcheson et al., 2012). The above findings and earlier reports have indicated that non-canonical TGF-β1 signaling may play a more substantial role in driving myofibroblast activation than canonical TGF-β1 signaling. Sildenafil has also been reported to block non-canonical TGF-β1 signaling in SSc fibroblasts as well (Higuchi et al., 2015) and also abrogate lipopolysaccharide (LPS)-induced proinflammation via downregulation of MAPK/nuclear factor kappa light-chain-enhancer of activated B cells (NFκB) signaling pathways in microglia cells (Zhao et al., 2011). Furthermore, sildenafil is reported to decrease Smad2/3 phosphorylation and ECM production in TGF-β1-treated cardiac fibroblasts of an animal model of pressure overload right ventricular hypertrophy (Rai et al., 2018). However, our study has not demonstrated the effect of sildenafil and SB204741 and their combination on canonical and non-canonical TGF-β1 signaling, which is the limitation of this study. Moreover, the above mechanism of inhibition of non-canonical and canonical TGF-β1 signaling by sildenafil, SB204741, and their combination might be a possible explanation for the amelioration of ACTA2 gene expression, leading to a decrease in ECM production as well as a decrease in the production of proinflammatory cytokines in our experimental model of PF (Figure 10).

Inflammation is one of the main pathological processes contributing to PF during long-term PD (de Lima et al., 2013). The characteristics of the inflammatory response include the expression of various cytokines and chemokines, as well as the infiltration of macrophages. Herein, in our study, we have reported the elevation of all proinflammatory cytokines upon culturing of HPFBs with TGF-β1. Another possible explanation of the involvement of 5-HT and TGF-β1 in the release of pro- and anti-inflammatory cytokines is its role in the regulation of T cells, which has been demonstrated in various studies (Wan et al., 2020). Th1 cells mainly produce IFN-γ, IL-2, and TNF-α; Th2 cells produce IL-4, IL-5, IL-6, IL-9, and IL-13; and Th17 cells produce IL-17A and IL-22 (Broos et al., 2016). IL-1β is reported to be released through the induction of other inflammatory cytokines (Tschopp et al., 2003). We tried to summarize the mechanisms and how immune cells are involved in the inflammatory process during peritoneal fibrosis (Supplementary Figure S6).

Furthermore, and in contrast to the rapid production of signature proinflammatory mediators, Tregs are known to dampen the inflammatory responses and promote wound repair through secretion of an immunosuppressive cytokine, IL-10 (Burmeister and Marriott, 2018). The role of 5-HT signaling in various diseases has been conflicting. In inflammatory bowel disease (IBD), rheumatoid arthritis (RA) and SSc blocking 5-HT signaling of macrophages and dendritic cells lessen the release of proinflammatory cytokines. IL-10 production was also enhanced in IBD with the inhibition of 5-HT signaling (Wan et al., 2020). However, in multiple sclerosis (MS), it has been demonstrated that 5-HT acts on T cells to produce less proinflammatory cytokines and more of IL-10 (Wan et al., 2020). In addition to the above findings, in our study, SB204741 also decreases proinflammatory cytokines. The above conclusion is in accordance with that of a previous study, in which a rat model of PF treated with KX2-391, a highly selective Src inhibitor, showed inhibited elevation of the above proinflammatory cytokines (Wang et al., 2017). Sildenafil is reported to exert its anti-inflammatory effect possibly through inactivated AMP-activated protein kinase (AMPK)-endothelial nitric oxide synthase (eNOS)/NO-NFκB [AMPK-eNOS/NO-NFκB] signaling (Rai et al., 2018). Administration of sildenafil has been reported in several studies to reduce the expression of IL-1β and TNF-α and increase the level of anti-inflammatory cytokine IL-10 (Raposo et al., 2013; Rai et al., 2018). Therefore, the prototypical anti-inflammatory cytokine, IL-10, expressed by monocytes, is reported to inhibit the expression of proinflammatory cytokines TNF, IL-1β, and IL-6 (Wang et al., 1994). In addition, sildenafil decreases the levels of various proinflammatory cytokines in the serum and bronchoalveolar lavage fluid (BALF), as well as oxidative and nitrosative stress in animal models of bronchial asthma (Laxmi et al., 2019). Herein, in this study, sildenafil, SB204741, and their combination reduced the production of proinflammatory cytokines in TGF-β1-induced HPFBs. Sildenafil alone and in combination resulted in the enhancement of the production of IL-10 (Figures 9G, H).

In our opinion, the above experiments’ data show, for the first time, that selective inhibition of type 5 of PDE enzymes, 5-HT_2B_ receptors, and their combination in a pre-treatment strategy compared to a post-treatment one could reverse the fibrotic phenotype of the HPFBs more effectively than in individual treatment by interfering with TGF-β1 ability of PMFB transformation.

In conclusion, this is the first study to demonstrate a synergistic potent anti-fibrotic effect of a combination of selective inhibitors of type 5 of PDE enzymes and 5-HT_2B_ receptor in an *in vitro* PF model. Furthermore, using a combination of these drugs could be considered for PD patients when PF is in the active phase for abrogating the EMT process of PMFBs, given that our data provide evidence of benefit. We also visualize that the combination will be more efficacious than using either of the drugs as a monotherapy. These results are likely to lead to further research leading to communication between the two canonical and non-canonical TGF-β1 pathways and also the development of novel therapeutic methods for the prevention and treatment of several fibrotic diseases.

## Limitations

Animal studies are needed for delineating the mechanism through which 5HT and TGF-β1 attenuate the peritoneal fibrosis. In addition, a large cohort of patients will strengthen the findings of the present study.

## Data Availability

The original contributions presented in the study are included in the article/Supplementary Material; further inquiries can be directed to the corresponding author.
