# Supplementary material for: Unravelling the role of Sildenafil and SB204741 in suppressing fibrotic potential of peritoneal fibroblasts obtained from PD patients
Source: Front Pharmacol. 2024 Jan 23;14:1279330. doi: 10.3389/fphar.2023.1279330 (PMC10844479; doi:10.3389/fphar.2023.1279330)
Supplement: Supplementary file 1 [file DataSheet2.PDF]

SUPPLEMENTARY TABLE S1. ***Primer Sequences***

	Primer Sequence, 5’-3’	
**Target Gene (Human)**	**Forward**	**Reverse**
COL1A1	CACACGTCTCGGTCATGGTA	AAGAGGAAGGCCAAGTCGAG
COL1A2	AGCAGGTCCTTGGAAACCTT	GAAAAGGAGTTGGACTTGGC
ACTA2	TCCTCATCCTCCCTTGAGAA	ATGAAGGATGGCTGGAACAG
CTGF	TGGAGATTTTGGGAGTACGG	TGGAGATTTTGGGAGTACGG
FN1	ACCTCGGTGTTGTAAGGTGG	CCATAAAGGGCAACCAAGAG
TIMP1	TTGACTTCTGGTGTCCCCAC	CTGTTGTTGCTGTGGCTGAT
MMP2	GGAAAGCCAGGATCCATTTT	ATGCCGCCTTTAACTGGAG
TGF-B1	GCCTTTCCTGCTTCTCATGG	TCCTTGCGGAAGTCAATGTAC
GAPDH	CAAGGTCATCCACGACCACT	CCAGTGAGTTTCCCGTTCAG
