# Supplementary material for: Unravelling the role of Sildenafil and SB204741 in suppressing fibrotic potential of peritoneal fibroblasts obtained from PD patients
Source: Front Pharmacol. 2024 Jan 23;14:1279330. doi: 10.3389/fphar.2023.1279330 (PMC10844479; doi:10.3389/fphar.2023.1279330)
Supplement: Supplementary file 2 [file DataSheet3.docx]

**Supplementary Methods**

**1. TGF-β1 standardization of dose**

HPFBs were cultured at 1x10^6^ cells/well in 6-well plates at 37°C in a humidified atmosphere containing 5% CO2. After overnight incubation, cells were grown in low serum DMEM (2% FBS containing DMEM). Later cells were washed and treated with medium only, media containing 2ng/ml, 5ng/ml, 10ng/ml and 20ng/ml of TGF-β1. The cells were treated for 24 hours and then lysed for total RNA extraction and mRNA expression by real-time PCR.

**2. MTT Assay**

To determine the toxicity of Sildenafil and SB204741 on the proliferative capacity of HPFBs, viability assay was performed. HPFBs were isolated using the above protocol mentioned and experiments were carried out between the passage 3 to 5. Briefly, HPFBs were seeded in 96-well plates at 20×10^3^ cells per well in DMEM growth medium with 10% FBS and incubated overnight at 37°C in a humidified environment containing 5% CO_2_. Culture medium from each well was discarded and replaced with new media on 3rd day. On 4th day, cells were synchronized with low serum medium for 24 hours. Following synchronization, fibroblasts were incubated with only media, media containing various concentrations of TGF-β1 (2ng/ml, 5ng/ml, 10ng/ml and 20ng/ml). Thereafter, TGF-β1 (10 ng/ml) was standardized and incubated with various concentrations of Sildenafil (1.0µM, 5.0µM, 10.0µM and 100µM) and of SB204741 (0.01µM, 0.1µM, 1.0µM, and 10.0µM) as well for 24 hours. Following incubation at 37°C in 5% CO_2_ for 24 hours, medium was discarded and 3-(4,5-dimethylthiazol-2-yl)-2,5- diphenyltetrazolium bromide (MTT) solution was added to each well (final concentration, 500 µg/ml) and incubated at 37°C for 3 hours. The supernatant was then removed and 150 µl of fresh DMSO was added to each well. Living cells reduce tetrazolium salt, which produces formazan crystal that is solubilized using DMSO. The absorbance of the solution was read at 570 nm with a microplate reader (Bio-Rad 550; Bio-Rad, Japan).

**3. Real-Time Polymerase Chain Reaction (RT-PCR)**

Total RNA was extracted from HPFBs using RNAiso Plus (Trizol method), and a total of 1μg of RNA was processed for cDNA synthesis using cDNA synthesis kit as per the manufacturer’s protocol. We performed real-time PCR reactions for each cDNA sample in triplicate using LightCycler® 480 2X Maxima SYBR Green Master Mix (Roche) and gene specific primer pairs for collagen type I alpha 1 chain (*COL1A1*), collagen type I alpha 2 chain (*COL1A2*), smooth muscle alpha (α)-2 actin (*ACTA2*), connective tissue growth factor (*CTGF*) and fibronectin1 (*FN1*) and tissue inhibitor of metalloproteinases1 (*TIMP1*), matrix metalloproteinase2 (*MMP2*), transforming growth factor beta1 (*TGF-Β1*) and glyceraldehyde 3-phosphate dehydrogenase (*GAPDH*) are listed in supplementary table 1. The PCR cycling was as follows: 50°C for 2 min for 1 cycle, 95°C for 10 min for 1 cycle, 95°C for 15 sec, 60°C for 1 min for 40 cycles in light cycler LC480 (Roche, USA). Gene expression was presented using a modification of the 2- Ct method. Relative fold difference between the groups was calculated by using the comparative cycle threshold (2^−ΔΔCt^) method. GAPDH was used as an endogenous control to normalize transcript levels of mRNA in each sample
