# Supplementary material for: Unravelling the role of Sildenafil and SB204741 in suppressing fibrotic potential of peritoneal fibroblasts obtained from PD patients
Source: Front Pharmacol. 2024 Jan 23;14:1279330. doi: 10.3389/fphar.2023.1279330 (PMC10844479; doi:10.3389/fphar.2023.1279330)

**Supplementary Figure S1**

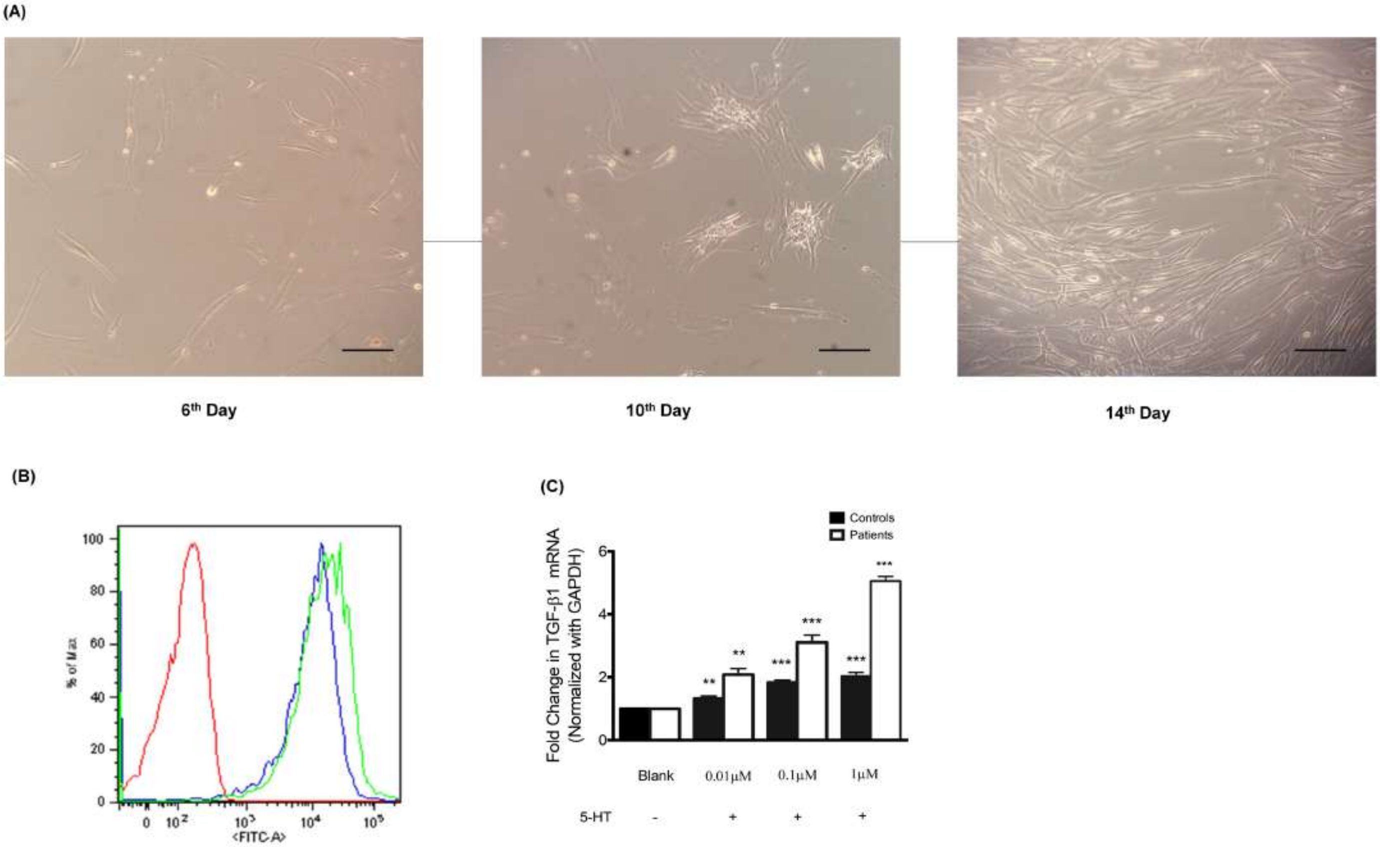

**Supplementary Figure S2**

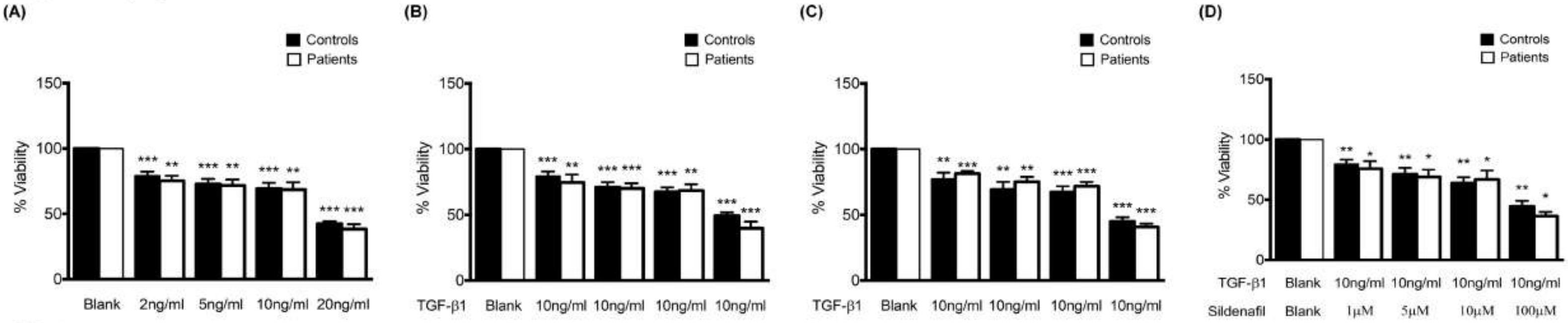

**Supplementary Figure S3**

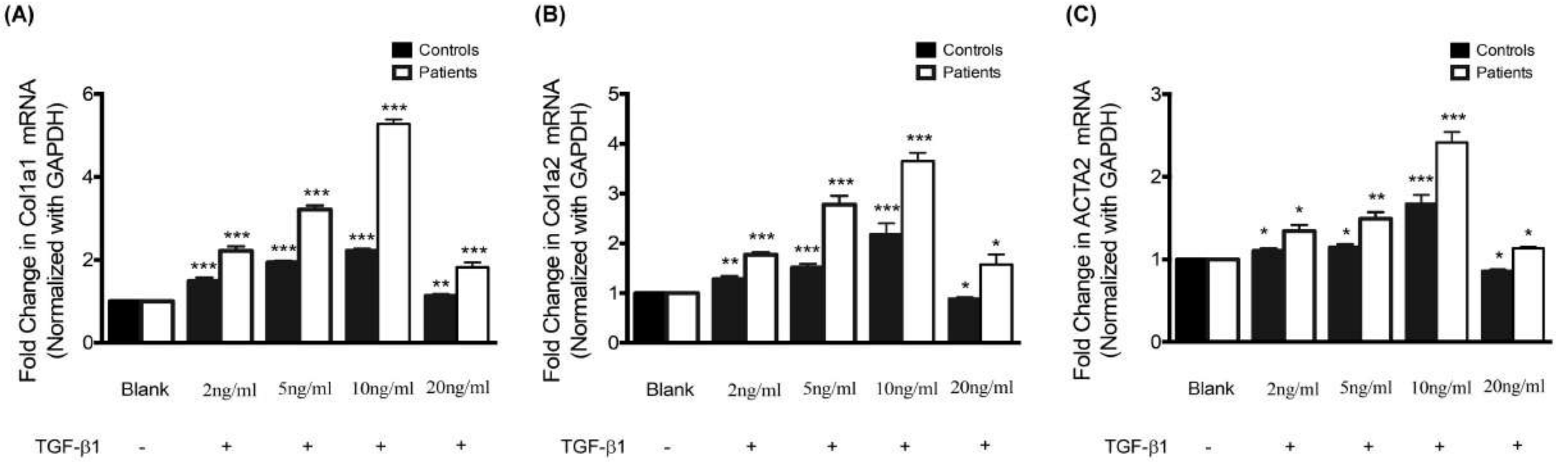

**Supplementary Figure S4**

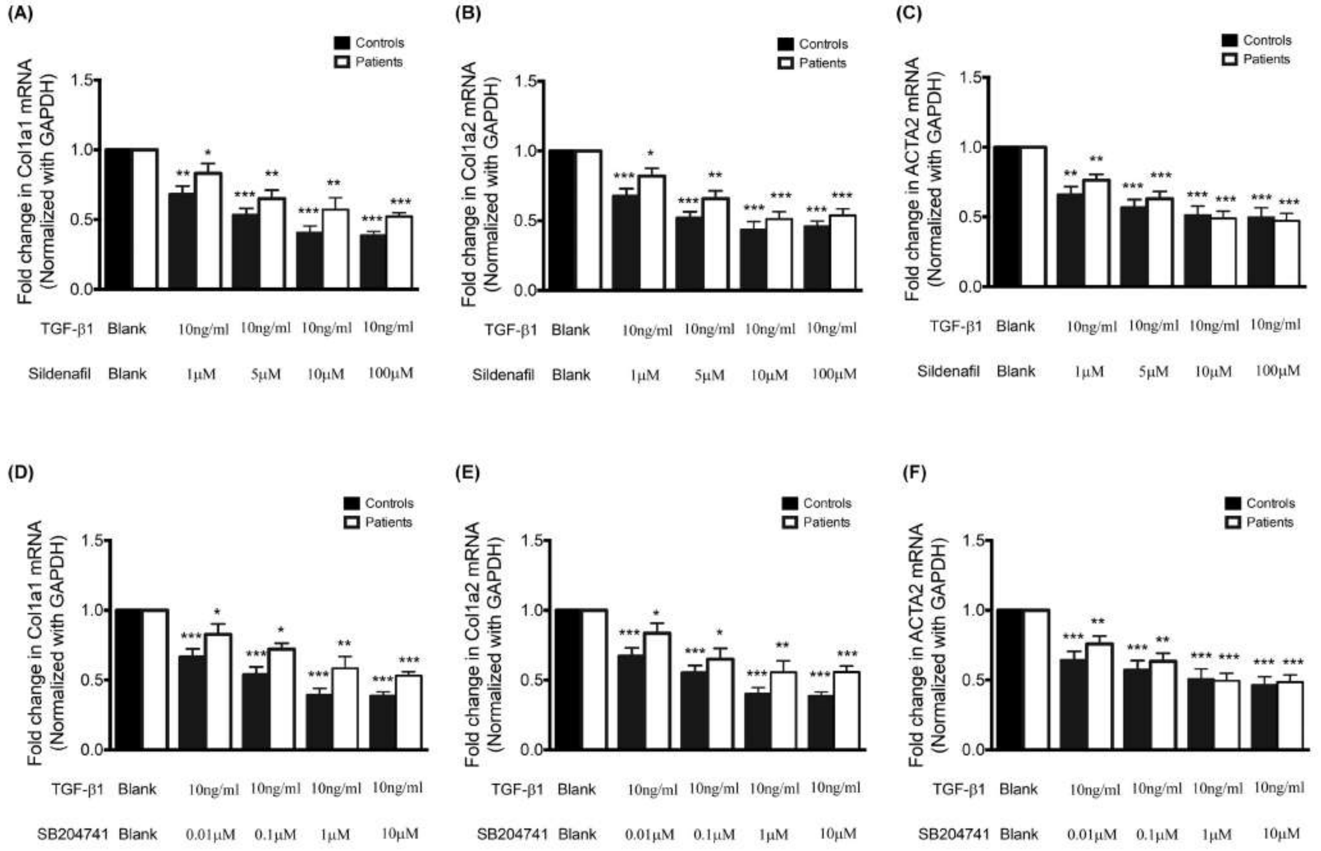

Supplementary Figure S5

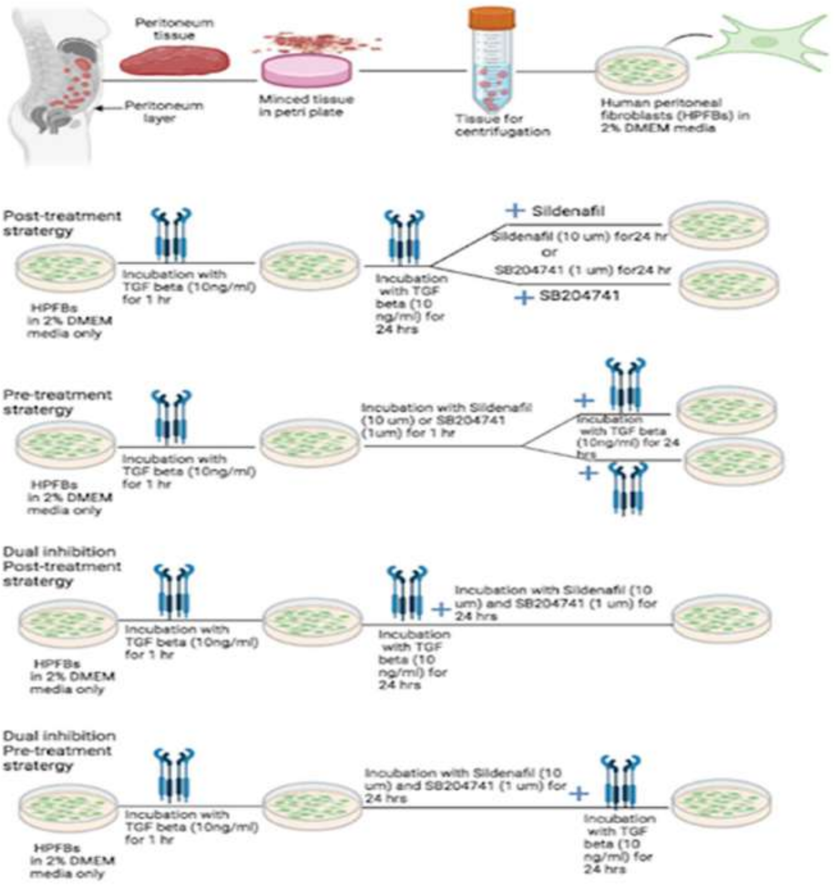

Supplementary Figure S6

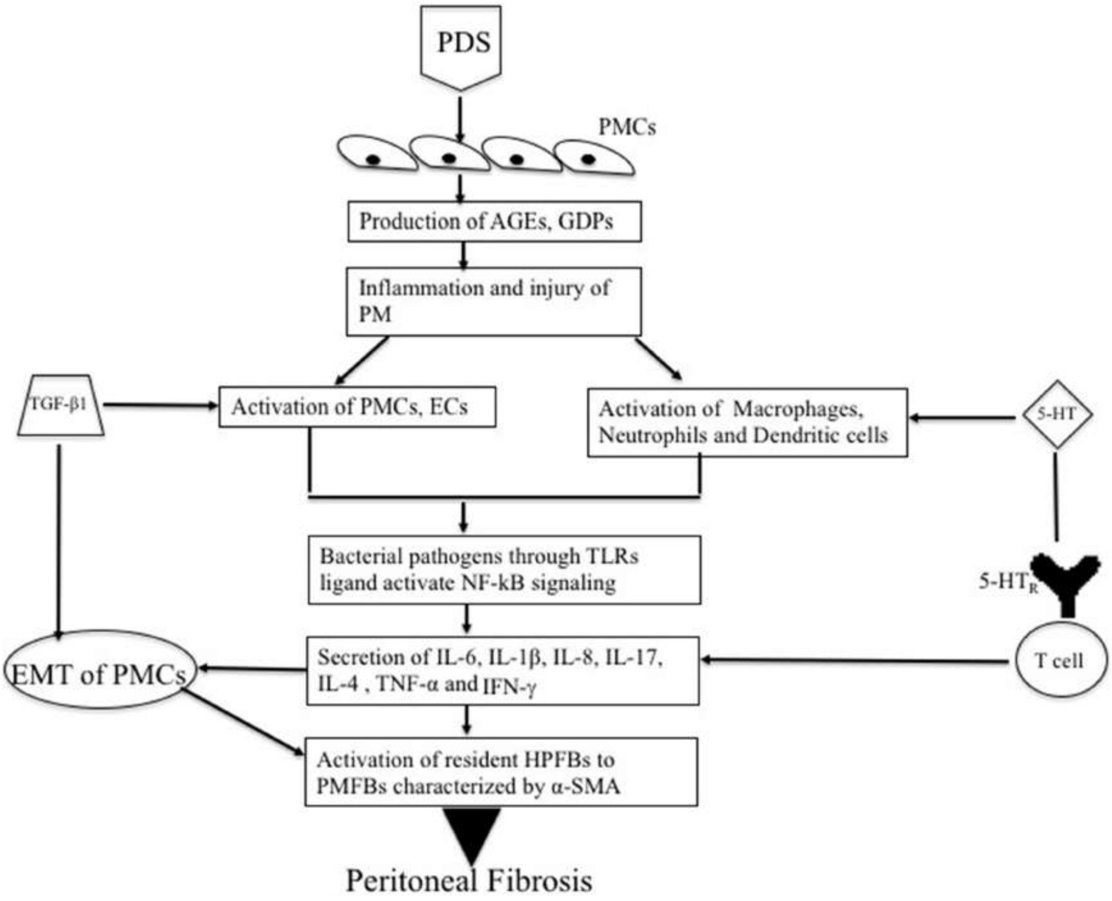

Supplement: Supplementary file 3 [file DataSheet1.PDF]
